# Cross-Species Multitask Learning with Molecular and ADME Descriptors for Liver Microsomal Metabolic Stability

**DOI:** 10.34133/csbj.0184

**Published:** 2026-08-11

**Authors:** Subhin Seomun, Sunyong Yoo

**Affiliations:** ^1^ Department of Intelligent Electronics and Computer Engineering, Chonnam National University, Gwangju, Republic of Korea.; ^2^ R&D Center, MATILO AI Inc., Gwangju, Republic of Korea.

## Abstract

Liver microsomal metabolic stability is a key determinant of *in vivo* exposure and an essential filter in lead optimization, yet cross-species prediction remains difficult because of heterogeneous metabolic pathways and limited model interpretability. We propose a cross-species multitask learning framework that integrates complementary molecular modalities—SMILES-derived fingerprints (Morgan and MACCS/RDKit), molecular graphs, and *in silico* absorption, distribution, metabolism, and excretion (ADME)/physicochemical descriptors—to predict binary microsomal stability (unstable: *t*_1/2_ ≤ 30 min; stable: *t*_1/2_ > 30 min) in human (HLM), rat (RLM), and mouse (MLM) liver microsomes. We curated 18,921 PubChem BioAssay measurements (6,685 HLM; 5,753 RLM; 6,483 MLM). Under stratified 10-fold Bemis–Murcko scaffold cross-validation with ensemble prediction and species-specific thresholds, the model achieved AUROC values of 0.811, 0.806, and 0.794 and AUPR values of 0.854, 0.860, and 0.862 for HLM, RLM, and MLM, respectively, consistently outperforming conventional machine-learning and single-task deep-learning baselines. SHapley Additive exPlanations (SHAP) identified transport/permeability indicators, CYP interaction flags, and the lipophilicity–polarity axis as the features most strongly associated with predicted stability. EdgeSHAPer, a graph neural network explanation method based on SHAP, highlighted stabilizing and destabilizing substructures. Recurrent destabilizing attributions were observed in alkene and allylic/benzylic contexts, whereas amide/carbamate motifs exhibited stabilizing attributions, with nitriles and halogens showing context-dependent effects. Fragment–ADME enrichment analysis characterized associations between local structural motifs and whole-molecule properties including lipophilicity, solubility, and blood–brain barrier permeability. This multi-modal, cross-species framework demonstrates that integrating structural encodings with ADME descriptors enhances both predictive performance and interpretability, yielding hypothesis-generating attributions for structural optimization that warrant prospective experimental validation.

## Graphical Abstract

**Figure:**
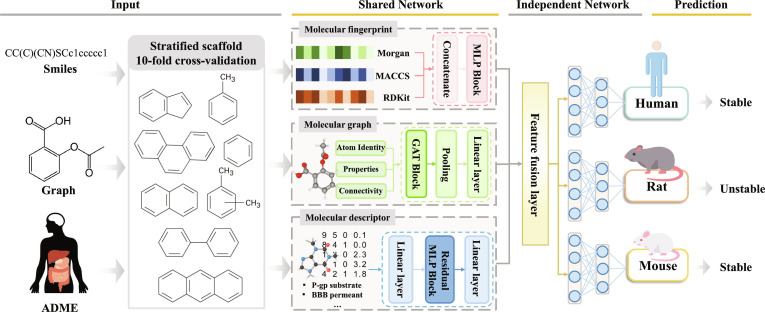

## Introduction

Liver microsomal stability is central to drug discovery, guiding lead optimization by reflecting biotransformation and transport relevant to *in vivo* exposure [1–4]. In practice, human (HLM), rat (RLM), and mouse (MLM) liver microsomal assays are frequently used in tandem to guide early structure optimization and to identify species differences that may complicate translation from discovery to preclinical studies [1,5]. Connecting fragment-level chemical destabilizing features—such as specific substructures prone to metabolic instability or toxicity—to whole-molecule absorption, distribution, metabolism, and excretion (ADME) descriptors remains challenging [6,7]. Establishing this connection is critical for rational drug design, as it enables medicinal chemists to identify and modify problematic substructures early in development while simultaneously predicting their impact on whole-molecule pharmacokinetic properties across species [8]. Moreover, metabolic stability reflects complex biotransformation pathways involving multiple enzymes and metabolic routes. This complexity limits the utility of simple empirical rules, motivating data-driven approaches [2,9,10].

Computational approaches have addressed this challenge through multiple strategies. MetStabOn developed species-specific models for human, rat, and mouse liver microsomal stability using ligand-based features to classify compounds into low-, medium-, and high-stability categories [11]. PredMS built a random forest (RF) binary classifier for HLMs (≥50% remaining at 30 min) and reported moderate performance on an external set [12]. CMMS-GCL integrates Simplified Molecular-Input Line-Entry System (SMILES)-based and graph-based encoders with contrastive learning to improve representation quality under data scarcity [13]. To address cross-species prediction, Long *et al.* [14] curated a large-scale HLM/RLM/MLM dataset and developed descriptor-based and graph neural network models. Using SHapley Additive exPlanations (SHAP) and atom-level attribution, they identified both shared and species-specific metabolic determinants, demonstrating the value of interpretable cross-species modeling. Collectively, these approaches have facilitated the prioritization of stable chemotypes. However, many existing models rely on single-modality structural encodings and single-species datasets. As a result, such models may underutilize complementary sources of information and often provide limited mechanistic insight into why a molecule is predicted to be stable or unstable [12,13,15]. Moreover, most frameworks do not explicitly integrate *in silico* ADME descriptors, such as permeability surrogates, drug-likeness rule indices, and enzyme-interaction flags, alongside structural encodings. This integration is essential for translating fragment-level modifications into predictions of whole-molecule pharmacokinetic behavior, yet remains underexplored in current approaches [13,15]. Finally, model interpretability is often summarized as molecular-level feature rankings, whereas direct attribution to chemically meaningful substructures and the alignment of these substructures with ADME descriptors across species remain less explored [16].

To address these gaps, we propose a cross-species multitask learning framework that integrates multi-modal molecular representations to predict liver microsomal stability. Specifically, the model leverages 3 complementary modalities: SMILES-derived fingerprints, molecular graphs, and *in silico* ADME descriptors. Multitask learning enables the model to exploit correlations among species while preserving species-specific metabolic differences [17]. These representations are processed within a shared network and species-specific independent networks, a design that captures generalizable structure–metabolism relationships while allowing species-dependent effects to be expressed through dedicated output layers. The model is trained and evaluated on a curated cross-species liver microsomal stability dataset from PubChem BioAssay [18]. This study has 2 main objectives. First, we seek to enhance the cross-species prediction of microsomal stability through a multitask learning framework that integrates structural and ADME features using multi-modal molecular representations. Second, we seek to identify substructures associated with each prediction and link them to ADME descriptors commonly used in medicinal chemistry.

## Materials and Methods

### Training dataset

The liver microsomal stability data were obtained from the PubChem BioAssay database [18]. We retrieved HLM, RLM, and MLM assays in which the standard type was reported as half-life (*t*_1/2_) and the experiments were performed in the absence of selective cytochrome P450 (CYP) isoform inhibitors [19,20]. For classification, compounds with *t*_1/2_ ≤ 30 min were labeled as unstable and those with *t*_1/2_ > 30 min were labeled as stable, following previous studies [1,21]; the 30-min cutoff falls near the mode of the per-species half-life distributions rather than in a sparse tail (Fig. S1). Because a nontrivial fraction of measurements are right-censored (“>30 min”; 17% to 22% per species, Table S1) or heavily rounded near the threshold (Fig. S2), a thresholded binary label is more robust than a continuous target. Relation-aware labeling, RDKit standardization, and within-species deduplication were performed using a uniform procedure across all datasets (Method S1). Cross-species label coverage is summarized in Table S2: of the 13,671 unique compounds, 64.9% were measured in only 1 species and just 3.4% in all 3. Because complete labels exist for only 3.4% of compounds, single-task per-species models cannot exploit information from compounds measured only in other species. We therefore adopt a multitask formulation in which a shared network learns from the full compound pool while species-specific independent networks capture metabolic profiles unique to each species. Missing species labels were masked during training (Method S2). After curation, the final dataset comprised 6,685 HLM, 5,753 RLM, and 6,483 MLM samples, whose per-species class composition is summarized in Table 1.

**Table 1.** Class composition of the PubChem training set: stable (*t*_1/2_ > 30 min) and unstable (*t*_1/2_ ≤ 30 min) compound counts per species (HLMs, RLMs, and MLMs), with totals

	Human (HLMs)	Rat (RLMs)	Mouse (MLMs)
Stable	2,758 (41.3%)	2,326 (40.4%)	2,418 (37.3%)
Unstable	3,927 (58.7%)	3,427 (59.6%)	4,065 (62.7%)
Total	6,685	5,753	6,483

HLMs, human liver microsomes; RLMs, rat liver microsomes; MLMs, mouse liver microsomes

### External evaluation dataset

An external evaluation set was assembled from ChEMBL, a data source independent of the PubChem BioAssay collection used for model development [22]. Human, rat, and mouse liver microsomal half-life (*t*_1/2_) measurements were retrieved from ChEMBL and processed through the uniform retrieval, relation-aware labeling, 30-min cutoff, RDKit standardization, and deduplication procedure described in Method S1; ChEMBL half-life values, reported in hours, were first converted to minutes, and discordant records within a compound–species pair were resolved by retaining the record with the most recent document year. Independence from the training data was then enforced at the structure level: every external compound was compared against the training set by RDKit canonical isomeric SMILES, and all compounds matching a training structure were removed, yielding a canonical-SMILES-disjoint external set of 906 compounds that provided 559 HLM, 235 RLM, and 286 MLM labeled measurements (Table 2). Because ChEMBL and PubChem sample partly overlapping drug-like chemical space, we characterized the structural relationship between the 2 sources (Fig. S3): the median nearest-neighbor Tanimoto similarity of external compounds to the training set was 0.71 (ECFP4), 110 compounds shared a desalted parent structure with a training compound, and a further 554 compounds carried a Bemis–Murcko scaffold absent from the training data. The external set is therefore source independent and disjoint at the canonical-SMILES level but not fully parent-disjoint, and this parent-level overlap is disclosed explicitly so that the degree of structural novelty is transparent. The same *in silico* ADME and physicochemical descriptors used for training were computed for all external compounds with SwissADME [23].

**Table 2.** Class composition of the independent ChEMBL external set: stable and unstable compound counts per species, with totals

	Human (HLMs)	Rat (RLMs)	Mouse (MLMs)
Stable	352 (63.0%)	126 (53.6%)	184 (64.3%)
Unstable	207 (37.0%)	109 (46.4%)	102 (35.7%)
Total	559	235	286

HLMs, human liver microsomes; RLMs, rat liver microsomes; MLMs, mouse liver microsomes

### Model overview

**Fig. 1.**
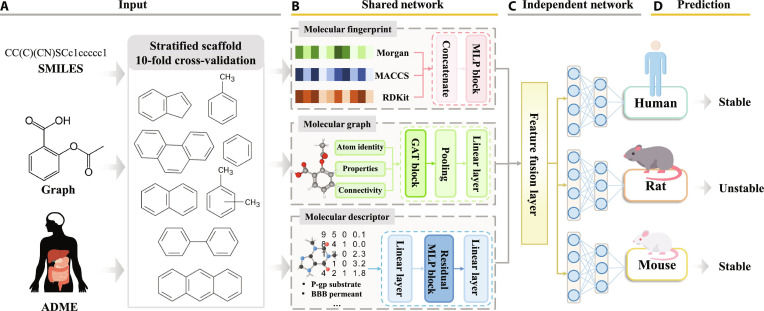
Overview of the proposed multi-modal, multitask framework for liver microsomal stability prediction. (A) Three molecular representations—Simplified Molecular-Input Line-Entry System (SMILES), molecular graph, and absorption, distribution, metabolism, and excretion (ADME) descriptors—serve as input. (B) A shared network processes fingerprints (Morgan, Molecular ACCess System [MACCS], and RDKit) through a multilayer perceptron (MLP) block, molecular graphs through a GATv2 block with pooling, and ADME/physicochemical descriptors through stacked linear layers and a residual MLP block and then combines the embedding in a feature fusion layer. (C) The fused representation feeds species-specific networks for humans, rats, and mice. (D) Each outputs a binary stable/unstable prediction.

The model predicts metabolic stability in HLMs, RLMs, and MLMs through a multi-modal, multitask architecture consisting of a shared network and species-specific independent networks (Fig. 1). Three molecular representations serve as input (Fig. 1A): SMILES-derived fingerprints comprising Morgan, Molecular ACCess System (MACCS), and RDKit; molecular graphs with node and edge features; and *in silico* ADME descriptors. In the shared network, modality-specific embeddings are generated through fingerprint concatenation, a graph attention block for graph processing, and multilayer perceptron (MLP) blocks for descriptor encoding (Fig. 1B). These embeddings are then concatenated through a feature fusion layer in the independent networks (Fig. 1C), where the fused representation is passed to species-specific networks to model individual metabolic characteristics. Finally, each species-specific network generates binary stability predictions (Fig. 1D). This architecture captures generalizable structural features through the shared network while modeling species-specific metabolic characteristics through dedicated independent networks.

### Molecular representation and embedding

#### Molecular fingerprint

The molecular structure is represented by 3 complementary 2-dimensional fingerprints: Morgan fingerprints [24], MACCS keys [25], and RDKit [26] topological fingerprint. Each fingerprint captures distinct aspects of molecular structure and connectivity. Morgan fingerprints encode circular atom neighborhoods by hashing local topologies, a representation widely used in structure–activity modeling. MACCS keys provide a 167-bit structural vector, where each bit indicates the presence or absence of a predefined substructure or functional group. This representation provides a compact, interpretable encoding that complements the hashed fingerprints [27]. RDKit topological fingerprints encode linear atom-bond paths of varying lengths, capturing connectivity patterns complementary to circular neighborhoods. For each molecule *x*, we compute the 2,048-bit Morgan *M*(*x*), 167-bit MACCS *A*(*x*), and 2,048-bit RDKit *R*(*x*) fingerprints and concatenate them into a single vector:

$$Z\left(x\right)=[M\left(x\right),A\left(x\right),R\left(x\right)].$$ (1)

Let *Z*(*x*) ∈ {0, 1}*^L^* denote the concatenated fingerprint vector. If any fingerprint is excluded, *L* is reduced by the corresponding bit length. To obtain a task-relevant representation, we apply a 2-layer MLP *f*(*Z*(*x*); *W*) to the fingerprint vector (see Table S3 for full hyperparameters).

$$f\left(Z\left(x\right);W\right)=\text{ReLU}\left(W_{2}\text{ReLU}\left(W_{1}Z\left(x\right)+b_{1}\right)+b_{2}\right).$$ (2)

*W*_1_ and *W*_2_ are weight matrices, *b*_1_ and *b*_2_ are bias vectors, and ReLU(*z*) = max(0,*z*). This process reduces dimensionality while retaining informative structure. The MLP layers enable nonlinear feature transformations that capture complex structural patterns.

#### Molecular graph

The molecular topology is encoded by representing each molecule as an undirected graph *G* = (*V*, *E*), where vertices represent atoms and edges represent covalent bonds. Each node is represented by a 77-dimensional feature vector encoding: atom type in 55 categories, degree in 7 levels, total hydrogens in 6 levels, formal charge in 3 levels, hybridization in 5 types, and aromaticity as one binary feature. We employed a graph attention module based on a graph attention network [28] to learn molecular graph representations. Rather than weighting neighbors by fixed degree-normalized coefficients, each layer learns attention coefficients for every edge from the features of the connected nodes together with their edge features and updates each node as an attention-weighted aggregation of its neighbors, so that the influence of each neighbor is learned dynamically rather than being fixed by node degree.

We stack 3 graph attention network layers with edge feature awareness (edge feature dimension 6), each layer using hidden dimension 256, 4 attention heads (64 dimensions per head, concatenated), and residual connections; each layer is followed by batch normalization, LeakyReLU(0.1) activation, and dropout (*p* = 0.35). Layer-wise representations are then aggregated by a Jumping Knowledge module in max mode to mitigate oversmoothing. Node-level features are pooled into a graph-level vector by concatenating global mean pooling and global max pooling, and the resulting (2 × 256)-dimensional vector is projected by a linear layer with LeakyReLU(0.1) to a 256-dimensional graph embedding used by the downstream fusion module (see Table S3 for full hyperparameters).

#### Molecular ADME and physicochemical descriptors

ADME descriptors were computed for each molecule using SwissADME [23]; all are derived from the molecular structure (SMILES) alone and use no experimental microsomal stability data. We organized them into numeric and categorical features. Numeric features comprise physicochemical and drug-likeness metrics, including molecular weight (MW), topological polar surface area (TPSA), lipophilicity estimates (iLOGP, XLOGP3, WLOGP [Wildman–Crippen log*P*], and MLOGP [Moriguchi log*P*]), heavy and aromatic atom counts, the fraction of sp^3^-hybridized carbons, the number of rotatable bonds, hydrogen-bond acceptor and donor counts, molar refractivity, and a skin permeation estimate. Additional features include violation counts for drug-likeness rules (Lipinski, etc.), bioavailability scores, pan-assay interference compounds and Brenk structural alerts, lead-likeness violations, and synthetic accessibility. Categorical features comprise gastrointestinal absorption, blood–brain barrier (BBB) permeability, P-glycoprotein (P-gp) substrate status, and inhibition predictions for 5 CYP isoforms (CYP1A2, CYP2C9, CYP2C19, CYP2D6, and CYP3A4); these were one-hot encoded, and missing numeric values were imputed during cross-validation (Method S3).

The ADME descriptors *x* were encoded by a residual MLP with skip connections [29]. After sanitization and normalization $\hat{x}=\mathrm{norm}\left(\text{sanitize}\left(x\right)\right)$ and a linear stem $h^{\left(0\right)}=\sigma \left(W^{\left(s\right)}\hat{x}+b^{\left(s\right)}\right)$, *L* residual blocks are applied:

$$h^{\left(l+1\right)}=h^{\left(l\right)}+G_{l}\left(h^{\left(l\right)}\right),\;l=0,\ldots ,L-1,$$ (3)

$$G_{l}\left(h\right)=W_{l}^{\left(2\right)}\sigma \left(W_{l}^{\left(1\right)}\mathrm{norm}\left(h\right)+b_{l}^{\left(1\right)}\right)+b_{l}^{\left(2\right)}.$$ (4)

This formulation preserves an unimpeded identity path while allowing each block to learn a bounded residual adjustment *G_l_*, which mitigates vanishing gradients and supports deeper compositions without relying on any particular normalization or activation choice. Subsequent to the composition of the *L* blocks, a linear head produces the descriptor embedding:

$$z=W^{\left(h\right)}\mathrm{norm}\left(h^{\left(L\right)}\right)+b^{\left(h\right)},$$ (5)

which serves as the final representation for downstream tasks. The resulting vector *z* is used for downstream fusion. We initialize linear weights with a Kaiming-normal scheme consistent with the chosen nonlinearity [30].

### Fusion layer

We fuse modality embeddings with a computationally efficient dimension-wise attention across modalities. Given a per-modality feature vector *x*, modality *m*, and optional bias *b*:

$$y=\sum_{m=1}^{M}\alpha_{m}x_{m}+b,$$ (6)

where each *α_m_* is a nonnegative per-dimension weight vector summing to one across modalities for each dimension. The weights are obtained by applying a softmax to learned modality scores and can optionally be interpolated with a uniform average through a scalar gate *g*: at *g* = 0, the fusion relies entirely on learned attention, and at *g* =1, it reduces to uniform averaging across modalities. Because the weights are dimension specific, different dimensions can emphasize different modalities. The fused vector is then passed to species-specific networks. Missing modalities and the detailed per-modality input handling—graph batching, dimension-aligned descriptor truncation and padding, and the zero-vector fallback that lets attention down-weight an absent modality—are described in Method S4.

### Prediction

Each species-specific prediction is generated by an independent network applied to the fused representation. Each head is a 3-layer block. The first layer applies LayerNorm(256) to the 256-dimensional fused vector and projects it via Linear(256, 128) with GELU activation and dropout (*p* = 0.35). The output layer is Linear(128, 1), producing a scalar logit for binary classification (see Table S3 for full hyperparameters). This design enables the model to learn shared multi-modal representations through the encoders and fusion module while maintaining species-specific decision boundaries through independent prediction heads.

### Loss function

To address class imbalance and multitask learning challenges, we implemented a composite loss combining focal loss and margin ranking loss. For each task *t*, the loss is

$$L_{t}=L_{\text{Focal}}+L_{\text{Ranking}},$$ (7)

where focal loss reduces the weight of well-classified examples and margin ranking loss encourages positive samples to have higher logits than negative samples. To balance task contributions dynamically, we applied uncertainty weighting with learnable parameters:

$$L_{\text{total}}=\sum_{t=1}^{3}\left(\frac{1}{2\sigma_{t}^{2}}L_{t}+\log\sigma_{t}\right).$$ (8)

Task-specific positive class weights were computed based on the training data distribution and clamped to the range (1, 20) to prevent extreme weighting. Because not every compound carried labels for all 3 species, missing labels were encoded as −1 and masked out of each task-specific loss prior to summation (see Method S2 for the masked-loss formulation).

### Training and evaluation

All models were trained and evaluated on the full curated dataset using a single, unified protocol: stratified scaffold-disjoint 10-fold cross-validation based on Bemis–Murcko scaffolds [31]. No separate train/test partition was used; the entire curated set entered the cross-validation, and scaffold-disjoint folds prevented leakage from structurally similar molecules while maintaining balanced class distributions across folds. All comparators—the conventional baselines (logistic regression [LR], RF, and support vector machine [SVM]), the single-modality and bimodal ablations, the fair single-task ablation, and the reimplemented CMMS-GCL and MS-BACL—were trained and evaluated under the same fold assignments, preprocessing, and optimization schedule as the proposed model, enabling fold-paired comparisons; the full baseline and ablation design, the unified within-fold preprocessing, and the verification that per-fold held-out sizes match in all 30 (species × fold) cells are detailed in Method S3 (per-test statistics in Table S4).

Descriptor imputation and standardization were fit on training-fold data only and applied unchanged to the held-out fold, preventing leakage of test-fold statistics into the descriptor pipeline (Method S3). Model parameters were optimized against the composite loss defined in the “Loss function” section using AdamW with cosine-annealing learning-rate scheduling and a linear warmup, gradient-norm clipping, and early stopping; the complete optimizer, scheduler, gradient-clipping, and early-stopping settings are listed in Table S3 and Method S5. Model performance was evaluated using area under the receiver operating characteristic curve (AUROC), area under the precision–recall curve (AUPR), Matthews correlation coefficient (MCC), F1-score, and accuracy; task-wise metric computation, including sigmoid conversion of logits, handling of degenerate label sets, and averaging across valid tasks, is detailed in Method S6.

### Baselines and ablation design

We benchmarked the proposed multitask multi-modal model against a set of comparators, all evaluated under the same stratified 10-fold scaffold cross-validation with identical fold assignments and preprocessing: conventional machine-learning baselines (LR, RF, and SVM) on the shared fingerprint features; single-modality deep-learning baselines (fingerprint-only, graph-only, and ADME-only); a fair single-task ablation matching one species-specific independent networks in architecture, modalities, capacity, and hyperparameters but without cross-species parameter sharing; 3 bimodal ablations (fingerprint [FP] + graph, FP + ADME, and graph + ADME); and 2 recent graph contrastive-learning methods, CMMS-GCL [13] and MS-BACL [15], reimplemented under the same scaffold-disjoint splits. Each comparator was contrasted with the proposed model using 2-sided paired Wilcoxon signed-rank tests on per-fold metrics with Benjamini–Hochberg false discovery rate (FDR) correction. Full construction details are provided in Method S3, and the statistical procedure in Method S7.

### SHAP and EdgeSHAPer for model interpretation

We employed SHAP [6] to quantify the magnitude and direction each ADME and physicochemical descriptor’s contribution to the per-species predictions [32]. SHAP values were estimated with KernelExplainer while the graph and fingerprint inputs were held at affixed background context so that only the descriptor vector varied, and features were ranked by mean absolute SHAP value; the background-sampling settings and the additive attribution formulation are given in Method S8.

Next, we applied EdgeSHAPer [7] to attribute each prediction to specific bonds, which were aggregated into molecular fragments and scored by a frequency-weighted mean contribution. We then examined fragment contributions and their consistency across species. The frequency-weighted aggregation, and the sign convention used for visualization, is defined in Method S9.

To assess fragment–ADME associations, molecules partitioned by fragment presence and tested for enrichment in continuous (effect size by Cohen’s *d*, significance by the Mann–Whitney *U* test [33]) and binary (log odds ratios [ORs], Fisher’s exact test [34]) ADME properties, with Benjamini–Hochberg control of the FDR; results are visualized as heatmaps of effect direction and magnitude, with markers denoting FDR-significant associations. Formal definitions of these measures and FDR-controlled testing procedure are provided in Method S10.

## Results

### Exploratory data analysis

An exploratory data analysis assessed data quality, split comparability, and chemical-space coverage prior to model training. Compounds were partitioned by Murcko scaffold so that all molecules sharing a core scaffold fall in the same subset [31], a widely used strategy that reduces the chance of close analogs spanning training and test sets and yields a more realistic estimate of generalization to novel scaffolds [35].

**Fig. 2.**
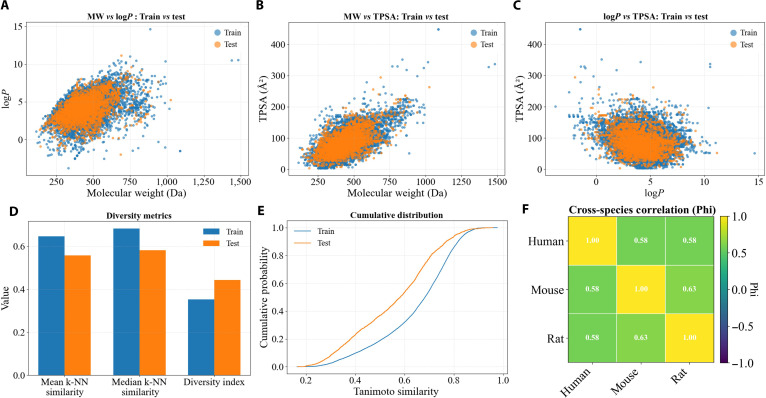
Exploratory data analysis of the scaffold-split liver microsomal stability dataset and cross-species label concordance. Overlaid scatter plots for key physicochemical pairs comparing the train (blue) and test (orange) sets: (A) molecular weight (MW) plotted against log*P*, (B) MW plotted against topological polar surface area (TPSA), and (C) log*P* plotted against TPSA. Distributions show substantial visual overlap between splits. (D) Mean and median *k*-nearest-neighbor (*k*-NN) Tanimoto similarity and a diversity index. (E) Empirical cumulative distribution functions (ECDFs) of *k*-NN Tanimoto similarity for the train and test sets, providing a bin-free view of nearest-neighbor similarity. (F) Cross-species correlation of binary labels summarized by the Phi coefficient.

Scatter plots of MW versus octanol–water partition coefficient (log*P*) (Fig. 2A), MW versus TPSA (Fig. 2B), and log*P* versus TPSA (Fig. 2C) showed largely overlapping training and test distributions, with systematic shift in MW, lipophilicity, or polarity; the few extreme-properties outliers (e.g., very high MW, log*P*, or TPSA) appeared in both subsets and likely reflect specialized chemotypes such as macrocycles or highly conjugated systems. The scaffold split therefore preserves comparable global chemical-space coverage across subsets.

*k*-nearest-neighbor (*k*-NN) Tanimoto similarities and a fingerprint diversity index (Fig. 2D and E) confirmed that test compounds are structurally more distinct: the mean *k*-NN similarity was lower for the test set than for the train set (mean *k*-NN 0.588 *vs* 0.673), with a correspondingly higher diversity index. Cross-species concordance, summarized as pairwise Phi coefficients (Fig. 2F), was moderately positive (human–mouse and human–rat ≈ 0.58; mouse–rat 0.63), indicating a shared structure–stability relationship despite species-specific metabolism (e.g., in CYP isoform expression and substrate preferences) that joint modeling can exploit, supporting multitask formulation. Together with the scaffold-disjoint construction of the split, these analyses indicate that performance under scaffold-disjoint cross-validation reflects generalization to structurally distinct scaffolds rather than recognition of close analogs.

### Performance evaluation

The proposed model was compared against conventional machine-learning baselines (LR, RF, and SVM), deep-learning single-modality baselines (fingerprint-, Graph-, or ADME-only), a fair single-task variant, and 2 recent graph contrastive-learning methods, CMMS-GCL [13] and MS-BACL [15]. All deep-learning models were trained and evaluated under stratified 10-fold scaffold cross-validation using identical fold assignments; CMMS-GCL and MS-BACL were additionally re-evaluated on our held-out test set under matched experimental conditions for direct comparability with the published values (Table 3).

**Table 3.** Ten-fold scaffold-disjoint cross-validation performance (AUROC and AUPR) per species for the proposed model, baselines, and ablations

	AUROC			AUPR		
Species	Human	Rat	Mouse	Human	Rat	Mouse
Model						
LR	0.617	0.636	0.584	0.688	0.719	0.693
RF	0.746	0.773	0.753	0.802	0.825	0.820
SVM	0.669	0.704	0.664	0.726	0.769	0.748
Single task	0.715	0.760	0.716	0.770	0.808	0.798
Fingerprint	0.725	0.748	0.711	0.778	0.780	0.780
Graph	0.626	0.650	0.560	0.693	0.707	0.726
ADME	0.614	0.660	0.603	0.685	0.737	0.721
FP + graph	0.722	0.741	0.718	0.775	0.800	0.794
Graph + ADME	0.633	0.677	0.617	0.698	0.748	0.723
FP + ADME	0.722	0.747	0.714	0.776	0.803	0.792
CMMS-GCL	0.685	0.716	0.688	0.744	0.774	0.775
MS-BACL	0.701	0.730	0.697	0.755	0.777	0.776
**Our model**	**0.811**	**0.806**	**0.794**	**0.854**	**0.860**	**0.862**

AUROC, area under the receiver operating characteristic curve; AUPR, area under the precision–recall curve; LR, logistic regression; RF, random forest; SVM, support vector machine; ADME, absorption, distribution, metabolism, and excretion; FP, fingerprint

The full model achieved the highest AUROC and AUPR for all 3 species and outperformed every comparator on the ranking metrics (2-sided paired Wilcoxon signed-rank tests across the 10 matched folds; all *q* ≤ 0.005 after Benjamini–Hochberg FDR correction; Table 3, Tables S4 and S5, and Figs. S4 and S5). On threshold-dependent metrics (MCC and F1), the advantage was species and metric dependent: the improvement was statistically significant for humans and mice but smaller and not uniformly significant for rats (paired Wilcoxon signed-rank tests with Benjamini–Hochberg correction; Table S4), where the single-task baseline is already strong (rat single-task AUROC = 0.760). We therefore frame the contribution as a robust, statistically supported gain in ranking microsomally unstable compounds, with a more nuanced advantage on classification-threshold-dependent measures.

Paired comparisons against each bimodal variant confirm that all 3 modalities contribute statistically significant, nonredundant signals: removing ADME (FP + graph) reduced AUROC by 0.065 to 0.089 across species; removing graph (FP + ADME), by 0.059 to 0.089; and removing fingerprints (graph + ADME), by 0.128 to 0.178 (all *q* < 0.005; Table S4 and Fig. S4). Restricting the descriptor block to closed-form physicochemical descriptors and rule-based medicinal-chemistry flags (“Deterministic-only”; Table S6) further separates the contribution of model-derived ADME predictions from that of pre-computed features. Fingerprints were the strongest single contributor, whereas graph and ADME descriptors contributed comparable, largely complementary signals, indicating that the graph branch is not redundant with fingerprints and supporting the use of all 3 modalities.

### External validation on an independent ChEMBL-derived dataset

We evaluated the trained model on the independent ChEMBL-derived external set described in the “External evaluation dataset” section (906 canonical-SMILES-disjoint compounds; Table 2). The proposed model achieved the highest AUROC among all evaluated methods for all 3 species—human (0.748), rat (0.756), and mouse (0.758)—and likewise the highest AUPR for human (0.769), rat (0.798), and mouse (0.801) (Table 4). As expected for evaluation across data sources, absolute performance was lower than under within-source scaffold cross-validation (AUROC 0.794 to 0.811; Table 3). We therefore read the external results as evidence that the model’s ranking and precision–recall performance both transfer to an independent experimental source across human, rat, and mouse microsomal stability, supporting cross-source portability.

**Table 4.** Performance (AUROC and AUPR) per species on the independent ChEMBL external set for the proposed model, baselines, and ablations

	AUROC			AUPR		
Species	Human	Rat	Mouse	Human	Rat	Mouse
Model						
LR	0.628	0.623	0.589	0.629	0.673	0.657
RF	0.701	0.736	0.712	0.726	0.784	0.769
SVM	0.644	0.654	0.644	0.635	0.670	0.676
Single task	0.736	0.740	0.732	0.750	0.770	0.781
Fingerprint	0.725	0.750	0.737	0.738	0.788	0.780
Graph	0.604	0.649	0.596	0.620	0.714	0.663
ADME	0.611	0.638	0.597	0.628	0.702	0.662
FP + graph	0.728	0.751	0.736	0.740	0.788	0.777
Graph + ADME	0.625	0.663	0.619	0.642	0.721	0.681
FP + ADME	0.730	0.749	0.739	0.744	0.786	0.775
CMMS-GCL	0.707	0.743	0.698	0.703	0.782	0.721
MS-BACL	0.703	0.741	0.715	0.699	0.772	0.731
**Our model**	**0.748**	**0.756**	**0.758**	**0.769**	**0.798**	**0.801**

AUROC, area under the receiver operating characteristic curve; AUPR, area under the precision–recall curve; LR, logistic regression; RF, random forest; SVM, support vector machine; ADME, absorption, distribution, metabolism, and excretion; FP, fingerprint

### SHAP-based interpretation of ADME descriptors for liver microsomal stability across species

**Fig. 3.**
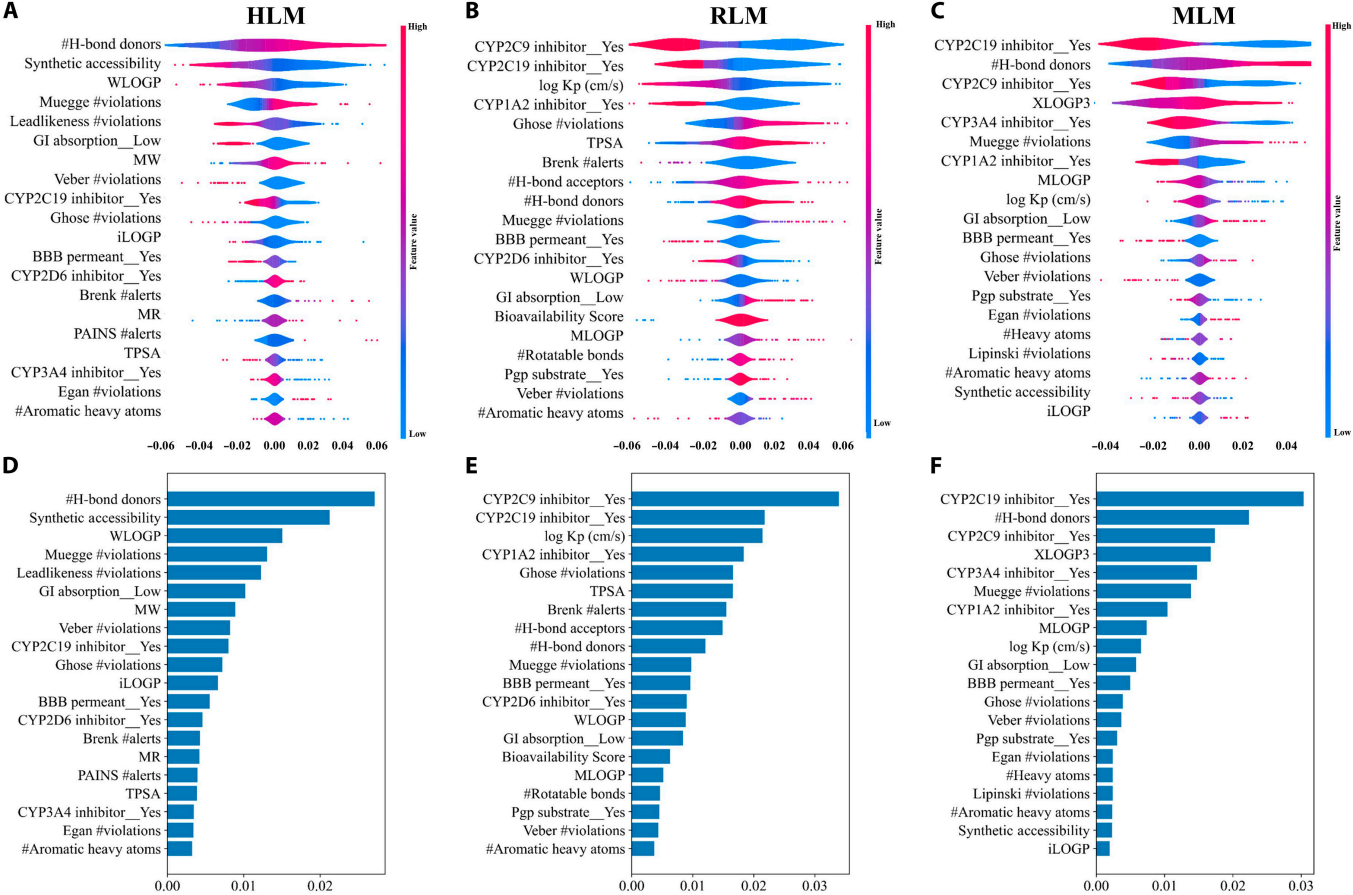
SHapley Additive exPlanations (SHAP)-based interpretation of absorption, distribution, metabolism, and excretion (ADME) descriptors across species. Beeswarm plots showing SHAP value distributions for molecular descriptors in (A) human (HLM), (B) rat (RLM), and (C) mouse liver microsomal (MLM) stability predictions. Each point represents a sample, with color indicating feature value (red, high; blue, low) and horizontal position showing the SHAP value (contribution to model output). Bar plots of the top 10 most important descriptors ranked by mean absolute SHAP value for (D) HLM, (E) RLM, and (F) MLM stability predictions.

We conducted a SHAP analysis of the liver microsomal stability model (Fig. 3). Throughout, positive SHAP values denotes the “unstable” class (*t*_1/2_ ≤ 30 min).

The beeswarm plots (Fig. 3A to C) reveal a polarity-associated instability pattern: higher-polarity features—high H-bond donor counts and TPSA—were consistently associated with the “unstable” class, consistent with established structure–metabolism relationships in which polar heteroatoms (e.g., hydroxyls and amines) serve as sites for phase I oxidation or orient substrates within the enzyme active site [36]. Conversely, lipophilicity-related metrics (log*P* family) generally contributed to stability; in this dataset, a relative lack of polar “metabolic handles” (H-bond donors) appears to be a stronger protective factor against rapid microsomal clearance [37].

Predicted inhibition of CYP2C9, CYP2C19, and CYP3A4 emerged among the top SHAP-attributed features [20], with compounds predicted to be inhibitors strongly associated with instability. This likely reflects shared binding-site recognition: substrates and competitive inhibitors often overlap in their CYP interactions, so compounds predicted as inhibitors may also undergo metabolic turnover [20,38]. Transport-related features (e.g., P-gp substrate and skin permeation coefficient [logKp]) further suggest that intracellular concentration and membrane permeability modulate enzymes access and thereby influence stability [39,40].

The mean absolute SHAP rankings (Fig. 3D to F) reveal interspecies differences in metabolic profiles. HLM predictions were dominated by physicochemical descriptors (H-bond donors, synthetic accessibility, and WLOGP), suggesting dependence on broad molecular properties rather than on the contribution of a single isoform, whereas RLM and MLM relied heavily on specific isoform interaction flags (e.g., CYP2C19 and CYP2C9 inhibition flags). This pattern is consistent with the known biological variation in CYP expression between humans and rodents, indicating that the multitask model recovered biologically meaningful species differences [41,42].

### Fragment-level explanations of metabolic stability per task

**Fig. 4.**
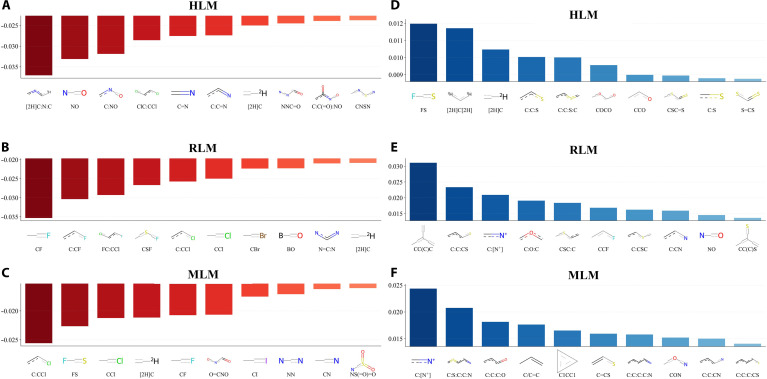
Fragment-level explanations of microsomal stability across species. Top 10 molecular fragments with the greatest impact on metabolic stability predictions. Bar plots showing bond-level SHapley Additive exPlanations (SHAP) contributions for destabilizing fragments (negative values, red bars) in (A) human (HLM), (B) rat (RLM), and (C) mouse liver microsomes (MLM) and stabilizing fragments (positive values, blue bars) in (D) HLM, (E) RLM, and (F) MLM stability. Representative molecular structures with highlighted bonds are shown below each bar.

We applied EdgeSHAPer to quantify each bond’s contribution to metabolic stability predictions on the held-out test set, using the resulting edge-importance scores (Method S9). Red fragments (Fig. 4A to C) indicate destabilizing features associated with decreased microsomal stability, and blue fragments (Fig. 4D to F), stabilizing features. Localizing these zones within individual molecules generates hypotheses for structural optimization that warrant prospective medicinal-chemistry validation.

Several fragment–outcome relationships recur across HLMs, RLMs, and MLMs under nicotinamide adenine dinucleotide phosphate (NADPH)-dependent microsomal oxidation. Alkenes and allylic/benzylic carbons were consistently attributed as destabilizing in all 3 species (Fig. 4A to C) [43,44]. Conversely, amide- and carbamate-containing carbonyl motifs appear on the stabilizing side under oxidative conditions (Fig. 4D to F) [45,46]. Other functionalities were context and species dependent: a vinyl nitrile context was associated with stabilization in RLMs (Fig. 4E), whereas a simple nitrile/CN fragment contributed to instability in MLMs (Fig. 4C) [47–49], suggesting that neighboring functionality and species-specific metabolism modulate the anticipated metabolic-masking effect. Sulfur functionalities showed oxidation-state-dependent behavior—thioethers and thiocarbonyls appearing on both sides, consistent with stepwise oxidation to sulfoxides/sulfones and, in some contexts, reactive-intermediate formation—and an S(VI) sulfonamide motif ranked among the most destabilizing features in MLM (Fig. 4C) [50,51]. Halogenation, although commonly used to block oxidative soft spots, played a position- and species-dependent role, with several halogenated fragments among the top destabilizing motifs in RLMs and MLMs (Fig. 4B and C) [47,52].

Under NADPH-dependent microsomal oxidation, recurrent fragment–outcome relationships emerge across HLMs, RLMs, and MLMs. Alkenes and allylic/benzylic carbons are consistently attributed as destabilizing features [43], consistent with CYP-mediated allylic C–H hydroxylation and C=C epoxidation that preferentially target these motifs and are often associated with clearance (Fig. 4A, C, and E) [48,53]. By contrast, nitrile groups (–C≡N) display pronounced scaffold and species dependence: a vinyl nitrile context associates with stabilization in RLM (Fig. 4E), whereas a simple CN fragment contributes to destabilization in MLM (Fig. 4F) [49,51]. These patterns suggest that neighboring functionality and species-specific metabolism may modulate the anticipated metabolic-masking effect. Sulfur-containing functionalities show oxidation-state-dependent behavior, with thioethers and thiocarbonyls appearing on both sides across species consistent with stepwise oxidation to sulfoxides/sulfones and, in certain contexts, the formation of reactive intermediates. In keeping with this propensity, an S(VI) sulfonamide motif is among the destabilizing features in MLM (Fig. 4C) [52]. Halogenation, although commonly used to block oxidative soft spots, shows a nuanced, position- and species-dependent role; several halogenated fragments rank among the top destabilizing motifs in RLMs and MLMs (Fig. 4B and C) [54]. Finally, carbonyl-containing motifs tend to correlate with stabilization under NADPH-dependent oxidative conditions (Fig. 4D to F) [45] while acknowledging that scaffold-specific hydrolytic pathways can predominate for particular chemotypes. Collectively, these patterns are consistent with a context- and species-dependent view of oxidative propensity and mitigation, underscoring the value of fragment-level interpretation for hypothesis-driven structural optimization.

We emphasize that SHAP and EdgeSHAPer values reflect feature-level contributions to the model output given the training data; they do not establish mechanistic causality or distinguish direct effects and effects mediated by correlated features. Several descriptor families used here—lipophilicity estimators, polarity surrogates, and molecular-size proxies—are strongly intercorrelated, so the attribution magnitude assigned to one feature may absorb a signal that biologically belongs to a correlated co-feature. Any SHAP-prioritized feature should therefore be confirmed through targeted experiments before being treated as a mechanistic determinant, and the fragment-level patterns above are best read as hypotheses for structural optimization rather than established determinants.

### Cross-species fragment–ADME enrichment in liver microsomes

**Fig. 5.**
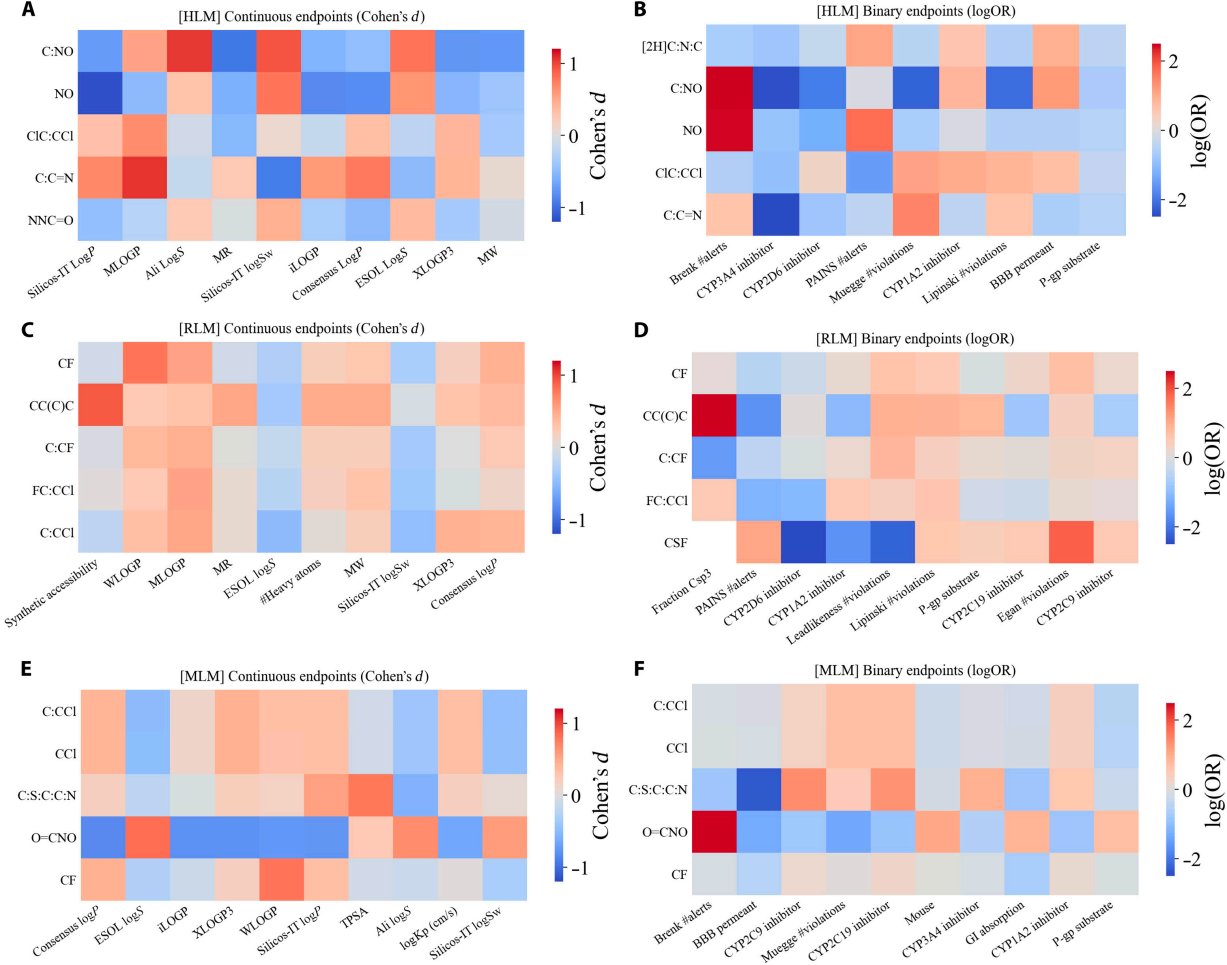
Fragment–absorption, distribution, metabolism, and excretion (ADME) enrichment across species. Heatmaps showing the association between top metabolic stability fragments and various ADME/physicochemical properties. Effect sizes for continuous endpoints (Cohen’s *d*) are displayed for (A) human liver microsomes (HLMs), (B) rat liver microsomes (RLMs), and (C) mouse liver microsomes (MLMs), while effect sizes for binary endpoints (log odds ratio) are shown for (D) HLMs, (E) RLMs, and (F) MLMs. Rows represent the top 10 EdgeSHAPer fragments identified for each species, and columns represent different ADME properties or molecular classifications.

Using the top 10 fragments highlighted by EdgeSHAPer, we mapped fragment–ADME associations across HLMs, RLMs, and MLMs and observed consistent medium-to-large effect sizes (Fig. 5A to F). Halogenated fragments, especially C–Cl and CF_3_-containing motifs, were associated with higher lipophilicity (consensus log*P*, XLOGP3, and WLOGP), greater molar refractivity, and lower predicted aqueous solubility (estimated solubility [ESOL] log*S*) (Fig. 5A to C) [55]. In several instances, these fragments were also enriched among compounds predicted as BBB-permeant (Fig. 5D to F) [56]. This enrichment pattern is chemically interpretable: halogenation, particularly CF_3_ substitution, typically increases lipophilicity and molar refractivity and can attenuate oxidative lability when substitution blocks oxidation-prone C–H positions [57,58]. The net impact on microsomal stability is context dependent. However, when overall polarity is approximately constant, increased lipophilicity combined with reduced oxidative susceptibility leads to greater passive membrane transport and, for some scaffolds, higher brain penetration. This aligns with the observed enrichment signal. Within the chemical space sampled by our PubChem-derived compounds, a higher predicted log-lipophilicity value tended to be associated with the stable class in the model’s attribution; this should be understood as a dataset-specific statistical pattern rather than a generalizable design rule (Fig. 5D to F) [56,57].

Fragments bearing amide or carbamate functionality showed the opposite trend, with a lower log*P*, a higher ESOL log*S*, and reduced predicted BBB permeation [59]. This is consistent with established BBB heuristics, which indicate that passive central nervous system (CNS) exposure declines as TPSA increases, particularly beyond about 60 to 90 Å^2^. The association also accords with the ESOL model, where log*P*, MW, aromaticity, and rotatable bond count are key predictors of solubility [60]. In addition to the polarity–permeability trade-off, several fragments previously identified as destabilizing aligned with canonical CYP-mediated oxidation motifs, most notably benzylic and allylic C–H bonds (Fig. 5D to F) [43,61]. These well-characterized metabolic hot spots provide a chemically plausible context for the unfavorable stability associations. In contrast, saturated or branched aliphatic fragments displayed ADME profiles that differed from flat, highly aromatic motifs, in line with the escape-from-flatland observation that increasing the sp^3^ content can improve developability (Fig. 5A to C) [62,63]. Finally, fragments with greater conformational flexibility and amphipathicity co-occurred with enrichment for P-gp substrates (Fig. 5D to F) [64], which is consistent with reports that P-gp preferentially recognizes hydrophobic, amphipathic scaffolds and actively exports them.

We interpreted effect sizes using pre-specified thresholds. For continuous endpoints, we highlighted fragments with the magnitude of Cohen’s *d* in the moderate-to-large range (Fig. 5A to C) [65]. For binary endpoints, we emphasized enrichments with |log(OR)| ≥ 0.69, which corresponds to OR ≥ 2 (Fig. 5D to F) [66]. The heatmaps are intended to aid pattern recognition and should be read as complements to the tables rather than substitutes [67]. Modest species-level differences are consistent with interspecies variation in hepatic enzyme expression across humans, rats, and mice [41].

Taken together, the enrichment analysis suggests several chemically plausible design hypotheses. Halogenation, particularly CF_3_ placement near oxidation-susceptible positions, increases lipophilicity and may enhance stability in some scaffolds by blocking vulnerable C–H sites and attenuating oxidative metabolism [68]. This benefit typically entails a predictable solubility penalty and, when polarity is held approximately constant, a greater likelihood of BBB permeation [69]. In contrast, carbonyl- and heteroatom-rich fragments tend to increase solubility and reduce BBB propensity at the expense of permeability, consistent with TPSA-based CNS heuristics and the ESOL framework [56]. Aromatic, allylic, and benzylic motifs are indicative of metabolic propensity, in line with established CYP chemistry (Fig. 5D to F) [43]. Increasing the sp^3^ character offers a practical countermeasure to these destabilizing features and can improve developability (Fig. 5A to C) [62]. Across species, these trends show limited divergence and provide actionable hypotheses for fragment substitution and placement when optimizing microsomal stability alongside broader ADME properties [70].

## Discussion

This study demonstrates a cross-species multitask framework that integrates complementary molecular representations—fingerprints, molecular graphs, and *in silico* ADME/physicochemical descriptors—to predict liver microsomal stability in humans, rats, and mice. Under scaffold-disjoint 10-fold cross-validation, the proposed model consistently outperformed conventional machine-learning baselines as well as single-task and single-modal deep-learning variants. In the single-modal comparison, ADME-only models and fingerprint-only models were individually competitive, whereas graph-only models were less consistent across species, supporting our interpretation that the gains arise from modality complementarity rather than any single dominant encoding.

To test whether this complementarity reflects genuine structure–activity learning rather than transfer of pre-computed ADME predictions, we performed a modality ablation under the same cross-validation (Table S6). The structural modalities alone recovered most of the full-model discrimination (AUROC 0.72 to 0.74 *vs* 0.80 to 0.81), and removing every model-derived ADME prediction—retaining only closed-form physicochemical descriptors and rule-based flags—reduced AUROC by approximately 0.04 to 0.07. Because the SwissADME predictors are computed solely from molecular structures and were trained on endpoints distinct from microsomal stability (e.g., CYP inhibition; PubChem AID 1851), this modest gain is more consistent with shared physicochemical signal than with leakage from the target endpoint. We cannot fully exclude compound-level overlap or shared medicinal-chemistry priors in the datasets used to develop these predictors, and therefore treat the descriptor block as a potentially informative prior whose contribution is quantified by ablation rather than claim complete independence.

The gain likely reflects 2 factors. First, the modalities capture distinct aspects of the structure–metabolism relationship—fingerprints encode the global substructure, graphs capture local bond environments, and descriptors summarize higher-level permeability- and transport-related properties—so the incremental improvement from the structural encoders indicates that context-dependent metabolic liabilities cannot be fully reduced to global properties. Second, multitask learning may act as regularizer, with shared parameters emphasizing cross-species associations, while task-specific heads model species-specific profiles. Consistent with the first factor, descriptor-level SHAP analysis highlighted the lipophilicity–polarity axis and transport/permeability indicators as the most prominent model-derived associations: higher polarity was associated with a higher predicted probability of instability and lipophilicity-related log*P* metrics were associated with stability, while CYP-inhibition flags (CYP2C9, CYP2C19, and CYP3A4) and transport descriptors (e.g., P-gp substrate and logKp) were associations with instability, suggesting that intracellular exposure may relate to enzyme access. These trends are chemically plausible but should be read as learned, hypothesis-generating associations rather than causal claims, since correlated features can redistribute attribution.

EdgeSHAPer localized bond-level effects: alkenes and allylic or benzylic contexts frequently appeared as destabilizing, whereas amide or carbamate motifs were more often associated with stability, and the context-dependent contributions of nitriles and halogens across species caution against overreliance on generalized empirical rules. Fragment–ADME enrichment further linked local edits to whole-molecule trade-offs—halogenated motifs were associated with higher lipophilicity, lower solubility, and increased BBB propensity, and amide-rich fragments were associated with the opposite trend—suggesting that stability optimization is better framed as a multi-objective balancing problem (jointly tuning lipophilicity and flexibility while shielding labile positions) than as isolated structural modifications.

Several limitations constrain these conclusions. Labels from heterogeneous PubChem BioAssay assays were binarized at a fixed half-life threshold. This ensures comparability with prior work and yields a robust, assay-agnostic label under interassay heterogeneity and censoring, but it compresses quantitative kinetic information: compounds near the threshold (e.g., 29 *vs* 31 min) are treated as maximally different, while compounds on the same side of the threshold (e.g., 31 *vs* 200 min) are treated identically, and it may amplify assay-level variability. A nontrivial fraction of compounds lie near the boundary (10% to 14% within ±5 min and 27% to 33% within ±15 min of the cutoff; Fig. S6A), and a substantial share of measurements are right-censored (“>30 min”) or heavily rounded (Fig. S2 and Table S1). Future work should therefore explore label formulations that retain more gradation: (a) ordinal classification into low/moderate/high-stability bins; (b) regression or censored-regression models (e.g., Tobit- or survival-style objectives) on harmonized assay subsets, ideally incorporating assay identifiers via hierarchical models to account for interassay variance; and (c) uncertainty-aware formulations—such as a near-threshold “gray zone” with label smoothing, conformal prediction, or evidential/Bayesian estimation—that flag low-confidence boundary predictions. Finally, *in silico* ADME descriptors inherit biases from their source models; augmenting them with experimental data and adding ensemble-based uncertainty quantification would further improve reliability in practical drug-discovery settings.

## Conclusions

This study demonstrates that a multi-modal, multitask learning framework effectively predicts liver microsomal stability across HLMs, RLMs, and MLMs with chemically interpretable attribution patterns. Descriptor-level SHAP analysis identified transport/permeability features, enzyme-interaction flags, and lipophilicity–polarity axis. EdgeSHAPer localized stabilizing and destabilizing substructures consistent with known metabolic soft spots and protective motifs. Performance was further evaluated on an independent ChEMBL-derived external dataset, supporting cross-source generalization for human, rat, and mouse microsomal stability. Fragment–ADME enrichment generates hypotheses for structural optimization that warrant prospective validation: adjusting lipophilicity and polarity, increasing sp^3^ content, blocking labile positions, and preserving stabilizing carbonyl motifs. A complete list of the abbreviations used throughout this article, together with their definitions, is provided in Table S7. Collectively, integrating structural and ADME representations within a cross-species framework enhances predictive performance and facilitates rational design to mitigate microsomal instability.

## Data Availability

The model implementation, the trained model, the inference and explanation software, and the preprocessed training and external-validation datasets are available at https://github.com/bmil-jnu/cross-species_metabolic_stability_prediction. A Google Colab notebook for running predictions without local installation is also provided in the repository.
