# Supplementary material for: Cross-Species Multitask Learning with Molecular and ADME Descriptors for Liver Microsomal Metabolic Stability
Source: Comput Struct Biotechnol J. 2026 Aug 11;35(1):0184. doi: 10.34133/csbj.0184 (PMC13457899; doi:10.34133/csbj.0184)
Supplement: Supplementary file 1 [file csbj.0184.f1.docx]

**Supplement**

**Supplementary Method S1. Data curation, relation-aware labeling, and deduplication**

The PubChem BioAssay training data and the ChEMBL external evaluation data were processed with a uniform curation and labeling pipeline; dataset-specific differences are noted explicitly below. Following retrieval of half-life assays measured without selective CYP-isoform-inhibitor co-incubation (main text Sections 2.1.1–2.1.2), continuous half-life values were normalized to minutes before the 30 min cutoff was applied (t½ ≤ 30 min, unstable; t½ > 30 min, stable). Inequality-qualified measurements (">", "≥", "<", "≤") were labeled only when the reported bound unambiguously determined the class relative to the cutoff — for example, a value ">30 min" implies stable and "<30 min" implies unstable — and boundary-ambiguous records were discarded. The relation-qualifier composition of the raw records and the effect of relation-aware versus numeric-only labeling are quantified in Supplementary Table S1 and Supplementary Figures S1, S2, and S6. Chemical structures were standardized with RDKit (neutralization and salt/largest-fragment removal); entries with missing or invalid SMILES were removed, and inorganic compounds were filtered out.

Records sharing the same non-isomeric canonical SMILES within a species were collapsed to a single representative selected by a fixed priority that favors the most direct evidence: a direct half-life measurement over a bound-derived label, and an exact relation ("=") over an inequality. Because this rule resolves any within-species label disagreement deterministically toward the most direct measurement, no separate averaging or majority-vote step was required. The three species datasets were curated independently, so a single compound could contribute up to three (compound, species) labels — one per species — without within-species label conflict. This convention was applied uniformly across all species and all models and therefore does not bias inter-model comparisons.

For the ChEMBL external set, records were deduplicated within each (compound, species) pair on the RDKit canonical isomeric SMILES. For compounds with multiple or discordant records in a species, a single representative label was selected by the most recent document year and, within the same year, by preferring direct half-life values over inequality-qualified ones.

**Supplementary Method S2. Multi-task loss masking**

Because not all molecules had labels for every species-specific endpoint, missing labels were encoded as −1 and excluded from loss calculation. For each molecule i and task k, the valid-label mask was defined as

$$m_{i,k}=\left\{ \begin{matrix} 1, & y_{i,k}\neq-1, \\ 0, & y_{i,k}=-1. \end{matrix} \right.$$

Here, *y_i,k_*$\in${0,1} denotes the binary label for molecule *i* and species-specific task *k*, and *z_i,k_* denotes the corresponding model logit. Invalid labels did not contribute to either the classification loss or the ranking loss.

For each task, class imbalance was handled using a focal classification loss computed only over valid labels. Let

$$p_{i,k}=\sigma\left( z_{i,k} \right),$$

where σ(·) is the sigmoid function, and let

$$p_{t,i,k}=y_{i,k} p_{i,k}+\left( 1-y_{i,k} \right)\left( 1-p_{i,k} \right).$$

The masked focal loss for task k was defined as

$$\mathcal{L}_{k}^{\mathrm{focal}}=-\frac{1}{\sum_{i} m_{i,k}}\sum_{i} m_{i,k} \alpha_{i,k}\left( 1-p_{t,i,k} \right)^{\gamma}\log\left( p_{t,i,k}+\epsilon\right),$$

where γ is the focal focusing parameter and ε is a small numerical constant. The class-weighting term *α_i,k_* incorporates the task-specific positive-class weight estimated from the training partition of each fold:

$$\alpha_{i,k}=\left\{ \begin{matrix} w_{k}^{+}, & y_{i,k}=1, \\ 1, & y_{i,k}=0. \end{matrix} \right.$$

The positive-class weight w_k_^+^ was computed from the training data distribution for task *k* and constrained to the range specified in the main Methods to prevent extreme weighting.

To encourage separation between positive- and negative-class compounds in logit space, we additionally applied a margin ranking loss within each task. For task *k*, let

$$\mathcal{P}_{k}=\{ i:m_{i,k}=1, y_{i,k}=1 \}$$

be the set of valid positive-class samples and

$$\mathcal{N}_{k}=\{ j:m_{j,k}=1, y_{j,k}=0 \}$$

be the set of valid negative-class samples in a mini-batch. The task-specific ranking loss was computed as

$$\mathcal{L}_{k}^{\mathrm{rank}}=\frac{1}{\left| \mathcal{P}_{k} \right| \left| \mathcal{N}_{k} \right|}\sum_{i\in\mathcal{P}_{k}} \sum_{j\in\mathcal{N}_{k}} \max\left( 0, \delta-\left( z_{i,k}-z_{j,k} \right) \right),$$

where δ is the margin. This term penalizes cases in which the positive-class logit is not higher than the negative-class logit by at least the specified margin. If a mini-batch did not contain at least one valid positive-class and one valid negative-class sample for a task, the ranking term for that task was set to zero.

The task-specific composite loss was then defined as

$$\mathcal{L}_{k}=\mathcal{L}_{k}^{\mathrm{focal}}+\lambda_{\mathrm{rank}} \mathcal{L}_{k}^{\mathrm{rank}},$$

where λ*_rank_* controls the contribution of the margin ranking term.

To balance the contribution of the three species-specific tasks, we applied uncertainty-based task weighting with learnable task parameters *s_k_*. The final multi-task objective was

$$\mathcal{L}_{\mathrm{total}}=\sum_{k=1}^{K} \left[ \exp\left( -s_{k} \right) \mathcal{L}_{k}+s_{k} \right],$$

where *K* = 3 for HLM, RLM, and MLM. This formulation allows the model to adjust the relative contribution of each task during training while retaining all valid labels available for each species.

Each task output was aligned with its corresponding label before the loss was computed. Samples with label −1 were removed from the task-specific loss terms through the valid-label mask. Tasks with no valid label in a given mini-batch were omitted from that update. In the rare case that a mini-batch contained no valid label for any task, a zero-valued placeholder loss was used so that optimization could proceed without interruption.

**Supplementary Method S3. Baselines and ablation design**

All comparators were evaluated under the same stratified 10-fold scaffold cross-validation as the proposed multi-task multi-modal model, using identical fold assignments and preprocessing to ensure like-for-like comparison.

**Conventional baselines.** Logistic Regression (LR), Random Forest (RF), and Support Vector Machine (SVM) were trained on the same fingerprint feature set used by the proposed model.

**Single-modality variants.** Three deep-learning baselines were each trained on a single modality — Fingerprint-only, Graph-only, and ADME-only — using the corresponding encoder of the proposed model together with a species-specific head per species.

**Fair single-task ablation.** A single species-specific branch was trained in isolation, matching the proposed model in architecture, modalities, capacity, and hyperparameters but with no cross-species parameter sharing. Because cross-species information can propagate only through shared parameters, joint optimization without shared parameters decomposes into independent per-species training; the single-task-versus-full-model contrast therefore isolates the contribution of cross-species sharing while controlling for architecture, modalities, capacity, and optimization.

**Bimodal ablations.** Three two-modality variants — FP+Graph (no ADME), FP+ADME (no Graph), and Graph+ADME (no Fingerprint) — were trained under the identical fold assignments and training procedure as the full model.

**External graph contrastive-learning methods.** CMMS-GCL and MS-BACL were re-implemented and re-evaluated under the same scaffold-disjoint 10-fold splits. Published hyperparameters were retained where reported, and preprocessing was unified across all models. The previously published held-out-only values for these methods are superseded by the cross-validated values in Table 3. The paired statistical comparison procedure used to contrast the proposed model against each comparator is detailed in Supplementary Method S7.

**Within-fold preprocessing.** Within each scaffold fold, numeric descriptor columns were imputed with the training-fold column median and standardized with a StandardScaler fit on the training fold; the fitted transformation was applied unchanged to the held-out fold. This procedure was identical for all models, so no test-fold statistics entered the descriptor pipeline.

**Per-fold size verification.** Held-out test-set sizes were verified to match those of the proposed model in all 30 (species × fold) cells; full per-test statistics, raw p-values, FDR-adjusted q-values, and effect sizes are reported in Supplementary Table S4.

**Supplementary Method S4. Model input handling and the multi-modal forward computation**

The model uses three molecular input modalities: graph structure, the fingerprint vector, and ADME/physicochemical descriptors. For each mini-batch, the graph input was represented as a PyTorch Geometric graph batch, and the concatenated fingerprint vector (Morgan, MACCS, and RDKit; the smil2vec field in the released implementation) was provided as a fixed-length vector. Descriptor vectors were provided as floating-point inputs and reshaped to the expected dimension where necessary. In all experiments reported here, every modality was present and every fingerprint and descriptor vector already matched the expected model input dimension, so no input was missing or required resizing. For robustness, the released implementation also includes defensive safeguards that were not exercised by the reported experiments: an unavailable modality would be replaced by a zero vector of the expected length, and a descriptor vector whose dimensionality did not match the expected input would be zero-padded or truncated to preserve dimensional consistency across samples. The model returned a shared pooled representation together with task-specific outputs; for classification, each task-specific output was interpreted as a logit and mapped to a probability with the sigmoid function when computing metrics.

**Supplementary Method S5. Optimization and gradient stabilization**

Beyond the optimizer and scheduler settings described in Section 2.3.6 of the main manuscript, the minimum learning rate in the cosine schedule was set to 1 × 10⁻⁶.

The gradient-clipping operation applied during training can be written as:

$g\leftarrow g\cdot min(1,\frac{c}{\left\| g \right\|_{2}})$, c = 1.0

where g denotes the concatenated gradient vector and c denotes the maximum gradient norm. The best-performing checkpoint identified by early stopping was retained for final evaluation in each scaffold fold.

**Supplementary Method S6. Evaluation metrics and task-wise averaging**

For each task, predicted logits were converted to probabilities using the sigmoid function:

$$\hat{p}_{i,k}=\sigma\left( z_{i,k} \right)=\frac{1}{1+exp(-z_{i,k})}$$

Evaluation metrics were calculated independently for each task after removing invalid labels and non-finite predictions. AUROC was computed only when both positive and negative classes were present. AUPR was calculated as the average precision or, alternatively, as the trapezoidal area under the precision–recall curve. Task-level scores were then averaged across available tasks using the mean of valid task scores.

For threshold-dependent metrics, predictions were binarized using a task-specific threshold when provided; otherwise, a default threshold of 0.5 was used. Reported threshold-dependent metrics comprise precision, recall, F1-score, Matthews correlation coefficient, accuracy, and specificity.

**Supplementary Method S7. Paired statistical comparison across cross-validation**

For each species s ∈ {human, mouse, rat} and each metric m ∈ {AUROC, AUPR, MCC, F1, Accuracy}, we compared the proposed model against every baseline and ablation using a two-sided paired Wilcoxon signed-rank test on the n = 10 per-fold values obtained from stratified 10-fold scaffold cross-validation. All comparators were trained and evaluated on identical fold assignments; the equality of per-fold test-set sizes across files was verified prior to pairing. Multiple-comparison correction was applied across the full family of 90 tests (6 comparators × 3 species × 5 metrics) using the Benjamini–Hochberg procedure to control the false discovery rate. Effect sizes were quantified as the matched-pairs rank-biserial correlation,

$$r_{rb}=\frac{\sum_{d>0}rank\left| d \right|-\sum_{d<0}rank\left| d \right|}{\sum rank\left| d \right|}$$

computed on the non-zero per-fold differences d = (proposed − comparator). Ninety-five percent confidence intervals across folds were computed as mean ± t · SE, where t is the 0.975 quantile of Student's t distribution with n − 1 = 9 degrees of freedom (t ≈ 2.262) and SE = SD/√n. Two interpretive caveats apply. First, per-fold metrics from k-fold cross-validation are not strictly independent because training folds overlap; we therefore used the non-parametric paired Wilcoxon test, which is more conservative than the paired t-test under such dependence. Second, with n = 10, the smallest attainable two-sided exact Wilcoxon p-value is 2 / 2¹⁰ = 1.95 × 10⁻³, so reported significant comparisons approach this lower bound; statistical significance is therefore interpreted jointly with the reported effect sizes and per-fold win/loss/tie counts.

**Supplementary Method S8. Descriptor-level SHAP analysis**

Descriptor-level interpretation was performed using SHAP KernelExplainer. To isolate the contribution of ADME and physicochemical descriptors, the graph and fingerprint inputs were held fixed at a reference molecular context, while only the descriptor vector was varied. Background and evaluation samples were selected by quantiles over model logits to cover a broad range of prediction scores.

For each task, SHAP values were computed as:

$$f\left( x \right)=\phi_{0}+\sum_{j=1}^{D} \phi_{j},$$

where *f(x)* denotes the model output for a given descriptor vector, $\phi$_0_ is the expected model output over the background distribution, and $\phi$_j_ is the contribution of descriptor *j*.

Feature importance was ranked by the mean absolute SHAP value across evaluated samples:

$$I_{j}=\frac{1}{N}\sum_{i=1}^{N} \left| \phi_{i,j} \right|$$

**[Background construction]**

Rather than random sampling or KMeans-based subsampling on the input space, we constructed the SHAP background by a logit-stratified mid-point quantile selection over the held-out fold. Specifically, a pool of candidate samples (256 by default) was drawn from the held-out fold of the cross-validation; for each pool sample, the model logit for the target task was computed under a fixed fingerprint and graph context (the fingerprint and graph features of the first pool sample), so that the only source of logit variation across the pool was the ADME descriptor block. The sorted logits were then divided into quantile bins (100 by default), and one representative was selected from the mid-point of each bin to form the background set. The evaluation set (256 samples by default) was selected by the same mid-point-quantile procedure with background indices excluded, so background and evaluation sets are disjoint by construction.

**[Why logit-stratified quantile selection]**

SHAP attribution magnitudes are defined with respect to the difference between an evaluation point and the background expectation, so a background concentrated in a narrow region of the model-output range would bias the attributions toward features that drive deviations from that region. Mid-point quantile selection over the logit distribution mitigates this bias by ensuring that the background expectation is taken over a representative cross-section of the model's prediction range. This is a stronger guarantee than uniform random sampling, which can over- or under-represent particular output regions, and then KMeans-based subsampling on the input feature space, which does not directly control for the distribution of model outputs.

**[Implementation parameters]**

For the analyses reported in the main manuscript, we used a candidate pool of 256 samples, a background set of 100 samples, an evaluation set of 256 samples, and 2,048 perturbation samples per evaluation point, with model inference performed in batches of 512. Because the fingerprint and graph contexts are held fixed at a reference sample during attribution, the reported SHAP values quantify the contribution of each ADME descriptor under that fixed structural context (denoted here the "fixed-context" attribution regime). For categorical descriptors that are one-hot encoded across multiple bits (for example, a CYP-inhibition flag encoded as a yes/no pair of bits), bit-level SHAP values can optionally be collapsed to a single descriptor-level value by summing or differencing the constituent bits; this collapsed form was used for the rankings reported in Section 3.3 and is available in the released implementation.

**[Sensitivity to background size]**

The logit-stratified design is intended to limit such sensitivity by construction, since the background expectation is anchored to a fixed cross-section of the model-output distribution rather than to a random subset that might shift between runs. We nevertheless acknowledge that an empirical sensitivity report is the more rigorous standard for descriptor rankings, and the released implementation supports repeating the analysis at additional background sizes so that the rank-correlation of the top-K feature rankings across background sizes can be reported as a stability check in follow-up work.

**[Reproducibility]**

All steps described above — pool construction, fixed-context logit computation, mid-point-quantile selection of the background and evaluation samples, application of the KernelExplainer with the specified perturbation budget, and the optional one-hot collapse — are implemented as parameterized routines in the released SHAP-analysis implementation. Default values are stated explicitly above; the same implementation can be used to reproduce the analysis or to repeat it under different parameter choices.

**Supplementary Method S9. EdgeSHAPer-based bond and fragment attribution**

EdgeSHAPer was used to estimate bond-level contributions to species-specific metabolic stability predictions. For each molecule, edge-level Shapley values were approximated under perturbations of molecular edges and aggregated to undirected bond-level attributions by summing the contributions of the two directed edges representing each bond. This procedure assigns an importance score to each bond, reflecting its contribution to the model output for the selected species.

Bonds were selected for downstream fragment analysis using a per-molecule fractional threshold rather than a fixed absolute cutoff. Within each molecule, bonds were ranked by the absolute value of their attribution and the top 20% (i.e., the 80th percentile of |attribution| within that molecule) were retained as non-negligible. A per-molecule fractional threshold was used because the absolute magnitude of an attribution depends on molecule size and on the model’s prediction confidence; fractional ranking preserves comparability across molecules of differing sizes and confidence levels. The fraction (default 20%) is a configurable parameter in the released implementation.

From the set of retained bonds within a molecule, an atom-level adjacency graph was constructed by joining the two atoms of every retained bond, and its maximal connected components were identified by depth-first search. Each connected component — a set of retained bonds linked through shared atoms — was treated as a single fragment and recorded by its atom and bond indices. Fragments are therefore defined by the joint retention pattern of their constituent bonds rather than by a predefined structural template such as Murcko scaffolds or BRICS fragments.

Each fragment was converted to a SMILES string with RDKit, using the molecule’s Kekulé form and RDKit’s default canonical atom ordering. Identical substructures from different molecules therefore map to the same string, which serves as the fragment identifier in the subsequent aggregation. Stereochemical centers within a fragment are retained, so two structurally identical fragments that carry different stereochemistry are treated as distinct identifiers; we did not perform stereochemical normalization beyond RDKit’s default behavior.

For fragments occurring in multiple molecules, the aggregated contribution was computed as a frequency-weighted mean of per-molecule mean attributions:

$$C_{r}=\frac{\sum_{i}s_{i,r}\cdot n_{i,r}}{\sum_{i}n_{i,r}}$$

where r indexes a fragment, i indexes molecules in which fragment r occurs, s_{i,r} is the average bond-level attribution of fragment r within molecule i, and n_{i,r} is the within-molecule occurrence count of r. Two alternative aggregations (an unweighted mean across molecules, and a sum) are also available in the released implementation. The frequency-weighted mean was retained as the default because it prevents fragments that appear in only one or two molecules from dominating the ranking while still allowing widely occurring fragments to be expressed proportionally to their frequency.

Aromatic rings were not pre-defined as atomic units. The connected-component step operates on the retained-bond subgraph alone, so an aromatic ring appears as a single fragment only when the per-bond attributions throughout that ring are jointly retained above the per-molecule threshold; otherwise the ring may appear as a partial sub-fragment. The procedure is applied identically across the three species (HLM, RLM, MLM): the same per-molecule fractional threshold, the same canonicalization, and the same aggregation rule are used, so any species-level differences reported in the manuscript reflect differences in learned bond attributions rather than differences in how fragments are extracted.

For consistency with the fragment-level figures in the main manuscript, the attribution sign was oriented so that positive fragment scores indicate stabilizing contributions and negative fragment scores indicate destabilizing contributions. Thus, positive fragment scores correspond to structural features that shift the prediction toward the stable class, whereas negative scores correspond to structural features that shift the prediction toward the unstable class. This sign convention is used only for fragment-level visualization and ranking. Like the SHAP-based descriptor attributions described in Method S8, the fragment-level outputs reported here represent model-derived associations and not experimentally validated mechanistic determinants.

**Supplementary Method S10. Fragment-ADME enrichment analysis**

Fragment-ADME enrichment analysis was conducted to connect local structural motifs identified by EdgeSHAPer with whole-molecule ADME and physicochemical properties. For each top-ranked fragment, molecules were partitioned into fragment-present and fragment-absent groups. Associations between fragment presence and continuous ADME descriptors were quantified using Cohen's d and tested using the Mann-Whitney U test:

$$d_{r,j}=\frac{\bar{x}_{r,j}^{present}-\bar{x}_{r,j}^{absent}}{s_{pooled}}$$

For binary ADME properties, such as predicted BBB permeability, gastrointestinal absorption, P-gp substrate status, or CYP inhibitor flags, enrichment was quantified using the log odds ratio and tested using Fisher's exact test:

$$\log\left( \mathrm{OR} \right)=log(\frac{ad}{cb})$$

where *a*, *b*, *c*, and *d* denote the cell counts in the 2 x 2 contingency table comparing fragment presence or absence with binary property presence or absence. Multiple testing was controlled using the Benjamini-Hochberg false discovery rate procedure. Heatmaps in the main manuscript visualize effect direction and magnitude, with statistical markers denoting FDR-significant associations when applicable.

**Supplementary Table S1. Relation-qualifier composition of the raw t½ records per species.** Counts of each measurement relation ("=", ">", "≥", "<", "≤") for HLM, RLM, and MLM. Right-censored records (">"/"≥") account for 21.6% (HLM), 19.8% (RLM), and 17.0% (MLM) of all measurements, and left-censored records ("<"/"≤") for a further ~2–4%. Because the exact half-life is undefined for these ~one-fifth of compounds, a thresholded (binary) label is more robust than a regression target; the table also explains the numeric-only vs relation-aware difference in Fig. S6B.

| **Species** | **<** | **<=** | **=** | **>** | **>=** | **All** |
| --- | --- | --- | --- | --- | --- | --- |
| **Human** | 11 | 2 | 5,196 | 1,439 | 22 | 6,771 |
| **Rat** | 150 | 0 | 4,525 | 1,154 | 2 | 5,831 |
| **Mouse** | 220 | 17 | 5,215 | 1,109 | 8 | 6,569 |

**Supplementary Table S2. Cross-species label coverage of the multi-task microsomal stability dataset.** Cross-species label coverage and compound overlap in the multi-task microsomal stability dataset. Section A reports per-species compound counts and class distributions after preprocessing (unstable: t½ ≤ 30 min; stable: t½ > 30 min). Section B summarizes pairwise compound overlap between species. Section C shows the number of unique compounds having a measured label in one, two, or all three species. Section D decomposes the total 13,671 unique compounds into non-overlapping Venn segments (e.g., HLM only = measured in human but not rat or mouse). Compounds with no label for a given species were assigned −1 and excluded from that species' loss during training. The high proportion of single-species compounds (64.9%) motivates the multi-task framework: the shared encoder provides cross-species regularization even when explicit label overlap is sparse, while species-specific heads capture idiosyncratic metabolic profiles. HLM, human liver microsomes; RLM, rat liver microsomes; MLM, mouse liver microsomes.

| **Category** | **Human (HLM)** | **Rat (RLM)** | **Mouse (MLM)** |
| --- | --- | --- | --- |
| **A. Per-species measurement summary** | | | |
| Total unique compounds | 6,685 | 5,753 | 6,483 |
| Unstable (t½ ≤ 30 min) | 2,758 (41.3%) | 2,326 (40.4%) | 2,418 (37.3%) |
| Stable (t½ > 30 min) | 3,927 (58.7%) | 3,427 (59.6%) | 4,065 (62.7%) |
| **B. Cross-species label overlap (pairwise)** | | | |
| **Species pair** | | **Shared compounds** | |
| HLM ∩ RLM | | 1,721 | (12.6% of 13,671) |
| HLM ∩ MLM | | 2,179 | (15.9%) |
| RLM ∩ MLM | | 1,808 | (13.2%) |
| **HLM ∩ RLM ∩ MLM** | | **458** | **(3.4%)** |
| **C. Compounds by number of species with a label** | | | |
| **Species coverage** | | **Compounds** | **% of total** |
| Measured in 1 species only | | 8,879 | 64.9% |
| Measured in 2 species | | 4,334 | 31.7% |
| **Measured in all 3 species** | | **458** | **3.4%** |
| **D. Exclusive species sets (non-overlapping Venn segments)** | | | |
| **Set** | | **Compounds** | **% of total** |
| HLM only | | 3,243 | 23.7% |
| RLM only | | 2,682 | 19.6% |
| MLM only | | 2,954 | 21.6% |
| HLM ∩ RLM only | | 1,263 | 9.2% |
| HLM ∩ MLM only | | 1,721 | 12.6% |
| RLM ∩ MLM only | | 1,350 | 9.9% |
| **HLM ∩ RLM ∩ MLM** | | **458** | **3.4%** |
| **Total unique compounds** | | **13,671** | **100%** |

**Supplementary Table S3. Architecture and optimization hyperparameters of the proposed cross-species multi-task multi-modal model.** Values are reported as used in the released implementation. Components correspond one-to-one with the modules described in Methods 2.3.1–2.3.6 of the main manuscript and with the corresponding components of the released implementation. "Dropout p" denotes the dropout probability; LayerNorm and BatchNorm1d denote standard layer and batch normalization, respectively. The fingerprint encoder concatenates Morgan (2048), MACCS (167), and RDKit (2048) bits prior to the input layer. The graph encoder applies GATv2 with edge-feature-aware attention; the attention heads are concatenated so that the per-layer output dimension equals the hidden dimension. The fusion module computes per-dimension attention weights normalized across modalities via softmax, with a scalar gate g interpolating between learned attention (g = 0) and uniform averaging (g = 1).

| **Component** | **Setting** |
| --- | --- |
| **Fingerprint encoder** | |
| Input dimension | Morgan 2048 + MACCS 167 + RDKit 2048 = 4263 |
| Architecture | Linear(4263, 256) → ReLU → BatchNorm1d → Dropout → Linear(256, 256) → ReLU → BatchNorm1d |
| Output embedding | 256 |
| Dropout p | 0.35 |
| Weight initialization | Kaiming-normal (fan-out, ReLU nonlinearity) |
| **Graph encoder** | |
| Convolution type | GATv2 (edge-feature-aware Graph Attention) |
| Node input dimension | 77 (atom features) |
| Edge input dimension | 6 (bond features) |
| Number of layers | 3 |
| Hidden dimension | 256 |
| Attention heads | 4 (concatenated, 64 dims per head) |
| Per-layer normalization | BatchNorm1d |
| Activation | LeakyReLU(0.1) |
| Dropout p | 0.35 |
| Residual connections | Yes (layer-wise) |
| Layer-wise aggregation | JumpingKnowledge (max) |
| Readout pooling | Concat(global mean, global max) |
| Output projection | Linear(2 × 256, 256) → LeakyReLU(0.1) |
| **Descriptor encoder** | |
| Architecture | Input LayerNorm → Stem [Linear(d, 256) → LeakyReLU(0.1)] → 3 residual blocks → Head [LayerNorm → Linear(256, 256)] |
| Residual block | LayerNorm → Linear(256, 256) → LeakyReLU(0.1) → Dropout → Linear(256, 256) + identity |
| Depth (residual blocks) | 3 |
| Width | 256 |
| Activation | LeakyReLU(0.1) |
| Normalization | LayerNorm (norm="ln") |
| Dropout p | 0.35 |
| Weight initialization | Kaiming-normal |
| **Fusion** | |
| Inputs | 3 views (fingerprint, graph, descriptor) × 256-dim each |
| Mechanism | Per-dimension softmax attention across modalities + scalar gate g interpolating with uniform mean |
| Per-branch projection | LayerNorm(256) before fusion (fp_norm, graph_norm, desc_norm) |
| Post-fusion | LayerNorm(256) → Dropout(0.35) |
| Output dimension | 256 |
| **Task heads** | |
| Number of heads | 3 (species-specific: HLM, RLM, MLM) |
| Architecture (per head) | LayerNorm(256) → Linear(256, 128) → GELU → Dropout(0.35) → Linear(128, 1) |
| Hidden width | 128 (= max(256/2, 64)) |
| Output | Scalar logit (binary classification per species) |
| **Optimization** | |
| Optimizer | AdamW |
| Learning rate | 1 × 10⁻⁴ |
| Weight decay | 1 × 10⁻² |
| Scheduler | Cosine annealing with linear warmup (first 5 epochs) |
| Gradient clipping | max norm 1.0 |
| Loss | BCE-with-logits + Focal Loss + Margin Ranking (uncertainty-weighted across tasks) |
| Maximum epochs | 200 (early stopping, patience 10 on held-out fold) |
| Cross-validation | Stratified 10-fold Bemis–Murcko scaffold splits |
| Threshold selection | Youden's J on held-out-fold predictions, per species |

**Supplementary Table S4. Paired statistical comparison of the proposed model against each baseline and ablation across the 10 matched scaffold cross-validation folds.** Each cell reports the mean per-fold difference Δ = (proposed − comparator) on the indicated metric for the indicated species. Positive Δ indicates that the proposed model outperformed the comparator on average across the 10 folds. Statistical significance is from two-sided paired Wilcoxon signed-rank tests with Benjamini–Hochberg FDR correction applied across the full family of 90 tests (6 comparators × 3 species × 5 metrics, including Accuracy not shown here): ** q < 0.01; * q < 0.05; ns q ≥ 0.05. Comparator labels follow the abbreviations defined in Methods 2.3.7. Full per-test statistics (raw p-values, FDR-adjusted q-values, win/loss/tie counts, matched-pairs rank-biserial effect sizes, including the Accuracy metric) are provided as Supplementary Data file paired_wilcoxon_results.csv. With n = 10 folds the minimum attainable two-sided exact p-value is 1.95 × 10⁻³.

| **Comparison** | **Species** | **AUROC** | **AUPR** | **MCC** | **F1** |
| --- | --- | --- | --- | --- | --- |
| **CMMS-GCL** | Human | +0.127 ** | +0.111 ** | +0.224 ** | +0.089 ** |
| **CMMS-GCL** | Mouse | +0.106 ** | +0.087 ** | +0.148 ** | +0.032 ** |
| **CMMS-GCL** | Rat | +0.090 ** | +0.086 ** | +0.137 ** | +0.045 * |
| **MS-BACL** | Human | +0.111 ** | +0.099 ** | +0.178 ** | +0.106 ** |
| **MS-BACL** | Mouse | +0.097 ** | +0.086 ** | +0.143 ** | −0.002 ns |
| **MS-BACL** | Rat | +0.076 ** | +0.083 ** | +0.091 ** | +0.035 ** |
| **Single-task** | Human | +0.096 ** | +0.084 ** | +0.127 ** | +0.071 ** |
| **Single-task** | Mouse | +0.078 ** | +0.064 ** | +0.082 * | +0.006 ns |
| **Single-task** | Rat | +0.045 ** | +0.051 ** | +0.014 ns | −0.009 ns |
| **FP+ ADME** | Human | +0.089 ** | +0.078 ** | +0.115 ** | +0.041 * |
| **FP+ ADME** | Mouse | +0.080 ** | +0.070 ** | +0.086 ** | +0.027 ns |
| **FP+ ADME** | Rat | +0.059 ** | +0.057 ** | +0.038 * | +0.024 ns |
| **Graph+ ADME** | Human | +0.178 ** | +0.156 ** | +0.248 ** | +0.136 ** |
| **Graph+ ADME** | Mouse | +0.177 ** | +0.139 ** | +0.222 ** | +0.179 ** |
| **Graph+ ADME** | Rat | +0.128 ** | +0.111 ** | +0.141 ** | +0.103 ** |
| **FP+Graph** | Human | +0.089 ** | +0.079 ** | +0.118 ** | +0.035 * |
| **FP+Graph** | Mouse | +0.076 ** | +0.061 ** | +0.073 ** | +0.028 ns |
| **FP+Graph** | Rat | +0.065 ** | +0.066 ** | +0.059 ** | +0.007 ns |

**Supplementary Table S5. Performance comparison across baselines, ablations, and the proposed model under 10-fold scaffold cross-validation (mean ± SD).** Best values per metric and species are shown in bold. Statistical significance versus the proposed model was assessed by two-sided paired Wilcoxon signed-rank tests on per-fold values with Benjamini–Hochberg FDR correction.

| **Species** | **Model** | **AUROC** | **AUPR** | **MCC** | **F1** | **Accuracy** |
| --- | --- | --- | --- | --- | --- | --- |
| **Human (HLM)** | Single task | 0.716 ± 0.032 ** | 0.771 ± 0.042 ** | 0.344 ± 0.054 ** | 0.711 ± 0.047 ** | 0.675 ± 0.030 ** |
|  | FP+Graph | 0.722 ± 0.022 ** | 0.775 ± 0.035 ** | 0.354 ± 0.036 ** | 0.747 ± 0.049 * | 0.690 ± 0.028 ** |
|  | Graph+ADME | 0.634 ± 0.024 ** | 0.699 ± 0.029 ** | 0.223 ± 0.031 ** | 0.646 ± 0.067 ** | 0.613 ± 0.021 ** |
|  | FP+ADME | 0.722 ± 0.020 ** | 0.777 ± 0.026 ** | 0.356 ± 0.036 ** | 0.741 ± 0.054 * | 0.690 ± 0.030 ** |
|  | CMMS-GCL | 0.685 ± 0.021 ** | 0.744 ± 0.034 ** | 0.247 ± 0.046 ** | 0.693 ± 0.057 ** | 0.636 ± 0.033 ** |
|  | MS-BACL | 0.701 ± 0.034 ** | 0.755 ± 0.040 ** | 0.293 ± 0.057 ** | 0.676 ± 0.069 ** | 0.648 ± 0.039 ** |
|  | **Our model** | **0.811 ± 0.001** | **0.854 ± 0.001** | **0.471 ± 0.007** | **0.782 ± 0.006** | **0.744 ± 0.004** |
| **Rat (RLM)** | Single task | 0.761 ± 0.023 ** | 0.809 ± 0.029 ** | 0.419 ± 0.046 ns | **0.767 ± 0.051** ns | **0.722 ± 0.029** ns |
|  | FP+Graph | 0.741 ± 0.026 ** | 0.794 ± 0.031 ** | 0.373 ± 0.045 ** | 0.751 ± 0.028 ns | 0.698 ± 0.023 ** |
|  | Graph+ADME | 0.678 ± 0.025 ** | 0.749 ± 0.031 ** | 0.292 ± 0.036 ** | 0.655 ± 0.121 ** | 0.634 ± 0.049 ** |
|  | FP+ADME | 0.747 ± 0.028 ** | 0.803 ± 0.017 ** | 0.394 ± 0.039 * | 0.734 ± 0.071 ns | 0.700 ± 0.037 ns |
|  | CMMS-GCL | 0.716 ± 0.031 ** | 0.774 ± 0.024 ** | 0.296 ± 0.060 ** | 0.713 ± 0.041 * | 0.656 ± 0.033 ** |
|  | MS-BACL | 0.730 ± 0.024 ** | 0.777 ± 0.025 ** | 0.342 ± 0.047 ** | 0.723 ± 0.030 ** | 0.679 ± 0.025 ** |
|  | **Our model** | **0.806 ± 0.001** | **0.860 ± 0.001** | **0.433 ± 0.005** | 0.758 ± 0.005 | 0.722 ± 0.004 |
| **Mouse**  **(MLM)** | Single task | 0.717 ± 0.037 ** | 0.799 ± 0.037 ** | 0.347 ± 0.063 * | 0.758 ± 0.039 ns | 0.697 ± 0.032 ns |
|  | FP+Graph | 0.718 ± 0.016 ** | 0.801 ± 0.015 ** | 0.357 ± 0.037 ** | 0.736 ± 0.071 ns | 0.686 ± 0.043 * |
|  | Graph+ADME | 0.617 ± 0.023 ** | 0.724 ± 0.021 ** | 0.208 ± 0.038 ** | 0.585 ± 0.075 ** | 0.575 ± 0.036 ** |
|  | FP+ADME | 0.714 ± 0.017 ** | 0.793 ± 0.020 ** | 0.344 ± 0.042 ** | 0.737 ± 0.055 ns | 0.684 ± 0.035 * |
|  | CMMS-GCL | 0.688 ± 0.023 ** | 0.775 ± 0.025 ** | 0.281 ± 0.047 ** | 0.732 ± 0.022 ** | 0.663 ± 0.022 ** |
|  | MS-BACL | 0.697 ± 0.030 ** | 0.776 ± 0.027 ** | 0.287 ± 0.064 ** | **0.766 ± 0.031** ns | 0.682 ± 0.030 ** |
|  | **Our model** | **0.794 ± 0.001** | **0.862 ± 0.001** | **0.430 ± 0.006** | 0.764 ± 0.007 | **0.721 ± 0.005** |

*Significance markers (vs. proposed model): ** q < 0.01, * q < 0.05, ns: not significant. With n = 10 folds, the minimum attainable two-sided exact Wilcoxon p-value is 2/2¹⁰ ≈ 1.95 × 10⁻³. Per-fold sample sizes were identical across all compared models, indicating shared scaffold partitions and validating fold-paired analyses.*

**Supplementary Table S6. Modality-ablation results under scaffold-disjoint 10-fold cross-validation.** “Deterministic-only” retains only closed-form physicochemical descriptors and rule-based medicinal-chemistry flags, with all model-derived ADME predictions removed.

| **Configuration** | **Species** | **AUROC** | **AUPR** | **MCC** |
| --- | --- | --- | --- | --- |
| **Full** | HLM | 0.811 ± 0.001 | 0.854 ± 0.001 | 0.471 ± 0.007 |
|  | RLM | 0.806 ± 0.001 | 0.860 ± 0.001 | 0.433 ± 0.005 |
|  | MLM | 0.794 ± 0.001 | 0.862 ± 0.001 | 0.430 ± 0.006 |
| **Deterministic-only** | HLM | 0.748 ± 0.019 | 0.800 ± 0.028 | 0.386 ± 0.030 |
|  | RLM | 0.761 ± 0.032 | 0.816 ± 0.028 | 0.405 ± 0.048 |
|  | MLM | 0.744 ± 0.022 | 0.819 ± 0.024 | 0.395 ± 0.048 |
| **Structural-only** | HLM | 0.722 ± 0.022 | 0.775 ± 0.035 | 0.354 ± 0.036 |
|  | RLM | 0.741 ± 0.026 | 0.794 ± 0.031 | 0.373 ± 0.045 |
|  | MLM | 0.718 ± 0.016 | 0.801 ± 0.015 | 0.357 ± 0.037 |

**Supplementary Table S7.** List of abbreviations used in the manuscript and their definitions.

| **Abbreviation** | **Definition** |
| --- | --- |
| ADME | absorption, distribution, metabolism, and excretion |
| AUPR | area under the precision–recall curve |
| AUROC | area under the receiver operating characteristic curve |
| BBB | blood–brain barrier |
| CNS | central nervous system |
| CYP | cytochrome P450 |
| ECCS | Extended Clearance Classification System |
| ECDF | empirical cumulative distribution function |
| EDA | exploratory data analysis |
| F1 | F1 score |
| FDR | false discovery rate |
| GAT | Graph Attention Network |
| GATv2 | Graph Attention Network, version 2 |
| HLM | human liver microsomes |
| k-NN | k-nearest neighbors |
| LipMetE | lipophilic metabolic efficiency |
| LogP | logarithm of the octanol–water partition coefficient |
| MACCS | Molecular ACCess System (keys) |
| MCC | Matthews correlation coefficient |
| MLM | mouse liver microsomes |
| MLP | multi-layer perceptron |
| MW | molecular weight |
| NADPH | nicotinamide adenine dinucleotide phosphate |
| OR | odds ratio |
| P-gp | P-glycoprotein |
| PAINS | pan-assay interference compounds |
| RLM | rat liver microsomes |
| SA | synthetic accessibility |
| SHAP | SHapley Additive exPlanations |
| SMILES | Simplified Molecular-Input Line-Entry System |
| TPSA | topological polar surface area |

**Supplementary Figure S1. Distribution of raw liver-microsomal half-life (t½) measurements per species (log scale).** Histograms of all curated record-level t½ values (in minutes, log10 x-axis) for human (HLM), rat (RLM), and mouse (MLM); the dashed line marks the 30 min classification cutoff. In all three species the cutoff lies near the mode of the distribution rather than in a sparse tail, indicating that the threshold separates the two most populated regions of the data rather than an arbitrary extreme. Values shown are raw per-species measurements prior to cross-species deduplication and final curation; the modeling dataset used for training is the reduplicated subset summarized in Fig. 1.

**Supplementary Figure S2. Fine-grained structure of t½ near the threshold (linear scale).** Top row (A–C): 0–60 min in 1-min bins for HLM, RLM, MLM. Bottom row (D–F): 0–5 min in 0.1-min bins. The dashed line marks 30 min. Two features are evident. (i) A pronounced pile-up exactly at 30 min (= 0.5 h) and at 60 min (= 1 h), together with a regular "comb" at integer-minute and round-hour values, reflects the limited precision and round-number reporting of the source assays. (ii) The 30 min pile is composed predominantly of right-censored ">30 min" records (57–78% of measurements in the 29–31 min window carry a ">" relation; highest in rat, ~78%; see Table S1), for which an exact half-life is not defined. Both properties argue against treating absolute t½ as a precise continuous target. Counts at exactly 1 min appear in the ~1 min bin (HLM 141, RLM 75, MLM 48 within ±0.1 min); the apparent 0 at the exact 1.000-min position reflects unit conversion from hours (1 min = 0.0167 h → 1.002 min after rounding).

**Supplementary Figure S3. Molecular-similarity relationship between the training (PubChem) and external (ChEMBL) datasets.** Nearest-neighbor Tanimoto similarity was computed on ECFP4 fingerprints (radius 2, 2048 bits) of desalted parent structures. **(A)** Density distribution of the maximum Tanimoto similarity of each external compound to the training set (orange; n = 906) and, for reference, the internal nearest-neighbor similarity within the training set (blue; n = 3,000 randomly sampled compounds, self-excluded). Dashed lines mark the medians (external 0.71; training 0.80). The external distribution is shifted toward lower similarity, indicating that external compounds are, on average, more structurally distant than a typical training compound; the spike at 1.0 corresponds to 244 external compounds with an ECFP4-identical nearest neighbor in training, of which 110 share the identical desalted parent structure. **(B)** Empirical cumulative distribution of the same similarities; 18% of external compounds have no training analog above Tanimoto 0.4 and ~50% fall below 0.7. Together these panels show that the ChEMBL external set, although drawn from a database independent of the PubChem training source, samples partly overlapping drug-like chemical space.

**Supplementary Figure S4. Cross-validated performance with 95% confidence intervals across stratified 10-fold scaffold cross-validation.** Mean values across the 10 folds are shown as points; horizontal bars denote 95% confidence intervals computed as mean ± t × SE, where t is the 0.975 quantile of Student's t distribution with n − 1 = 9 degrees of freedom (t ≈ 2.262). Rows correspond to AUROC, AUPR, MCC, and F1; columns correspond to Human (HLM), Mouse (MLM), and Rat (RLM). Models compared are the proposed full model ("Our model"), two literature baselines re-evaluated under the identical scaffold splits (CMMS-GCL, MS-BACL), the single-task ablation (per-species networks with the identical architecture and hyperparameters as a single branch of the full model; equivalent to a multi-task model without shared layers, see Methods §2.3.7), and three modality ablations (FP + Graph; FP + ADME; Graph + ADME). Numerical values printed above each marker are the per-species fold means. All models were trained and evaluated on identical fold assignments. Per-test statistics and effect sizes are reported in Table S4.

**Supplementary Figure S5. Per-fold performance distributions and paired Wilcoxon signed-rank tests against the proposed model.** Boxes denote the interquartile range with the median line; whiskers extend to 1.5 × IQR; per-fold values are overlaid as jittered points (n = 10 folds per species per model). Rows: AUROC, AUPR, MCC, F1; columns: Human (HLM), Mouse (MLM), Rat (RLM). Significance brackets compare each baseline or ablation to the proposed full model using two-sided paired Wilcoxon signed-rank tests over the 10 matched scaffold folds, with Benjamini–Hochberg FDR correction applied across the full family of 90 tests (6 comparisons × 3 species × 5 metrics). Markers: ** q < 0.01, * q < 0.05, ns q ≥ 0.05. With n = 10, the minimum attainable two-sided exact p-value is 2 / 2¹⁰ = 1.95 × 10⁻³, so all significant comparisons reported here approach this lower bound. Models and abbreviations as in Figure S4. Full test statistics, raw p-values, FDR-adjusted q-values, and matched-pairs rank-biserial effect sizes are tabulated in Table S4.

 **Supplementary Figure S6. Near-threshold mass and effect of relation-aware labeling (raw record level).** (A) Percentage of compounds whose t½ falls within ±5 min (25–35), ±10 min (20–40), and ±15 min (15–45) of the 30 min cutoff. A non-trivial fraction sits near the boundary: ±5 min, HLM 10.2% / RLM 13.6% / MLM 10.6%; ±15 min, HLM 26.7% / RLM 32.6% / MLM 28.8%. Excluding the ±5 min window changes the (relation-aware) stable fraction by ≤1.5 percentage points in every species, indicating that boundary compounds do not drive the overall class balance. (B) Stable fraction computed with numeric-only versus relation-aware labeling. Respecting the censoring relation (e.g., treating ">30 min" as stable) increases the stable fraction by +2.3 (HLM), +5.7 (RLM), and +2.2 (MLM) percentage points; the larger rat shift reflects its higher proportion of ">30 min" right-censored records (Table S1).
